# STING agonist diABZI induces PANoptosis and DNA mediated acute respiratory distress syndrome (ARDS)

**DOI:** 10.1038/s41419-022-04664-5

**Published:** 2022-03-25

**Authors:** Yasmine Messaoud-Nacer, Elodie Culerier, Stéphanie Rose, Isabelle Maillet, Nathalie Rouxel, Sylvain Briault, Bernhard Ryffel, Valerie F. J. Quesniaux, Dieudonnée Togbe

**Affiliations:** 1 CNRS -UMR7355, 45071 Orleans Cedex 2, France; 2 Experimental and Molecular Immunology and Neurogenetics, University of Orleans, Rue de la Ferollerie, 45071 Orleans, Cedex 2 France; 3 Artimmune SAS, 13 Avenue Buffon, 45100 Orleans, Cedex 2 France; 4 Genetics department, Regional Hospital Orleans (CHRO), Orleans, 45100 France

**Keywords:** Cell death and immune response, Respiratory tract diseases

## Abstract

Stimulator of interferon genes (STING) contributes to immune responses against tumors and may control viral infection including SARS-CoV-2 infection. However, activation of the STING pathway by airway silica or smoke exposure leads to cell death, self-dsDNA release, and STING/type I IFN dependent acute lung inflammation/ARDS. The inflammatory response induced by a synthetic non-nucleotide-based diABZI STING agonist, in comparison to the natural cyclic dinucleotide cGAMP, is unknown. A low dose of diABZI (1 µg by endotracheal route for 3 consecutive days) triggered an acute neutrophilic inflammation, disruption of the respiratory barrier, DNA release with NET formation, PANoptosis cell death, and inflammatory cytokines with type I IFN dependent acute lung inflammation. Downstream upregulation of DNA sensors including cGAS, DDX41, IFI204, as well as NLRP3 and AIM2 inflammasomes, suggested a secondary inflammatory response to dsDNA as a danger signal. DNase I treatment, inhibition of NET formation together with an investigation in gene-deficient mice highlighted extracellular DNA and TLR9, but not cGAS, as central to diABZI-induced neutrophilic response. Therefore, activation of acute cell death with DNA release may lead to ARDS which may be modeled by diABZI. These results show that airway targeting by STING activator as a therapeutic strategy for infection may enhance lung inflammation with severe ARDS.

**Figure:**
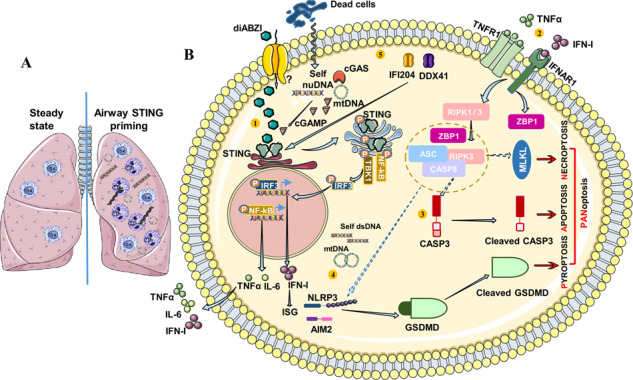
STING agonist diABZI induces neutrophilic lung inflammation and PANoptosis **A**, Airway STING priming induce a neutrophilic lung inflammation with epithelial barrier damage, double-stranded DNA release in the bronchoalvelolar space, cell death, NETosis and type I interferon release. **B**, **1.** The diamidobenzimidazole (diABZI), a STING agonist is internalized into the cytoplasm through unknown receptor and induce the activation and dimerization of STING followed by TBK1/IRF3 phosporylation leading to type I IFN response. STING activation also leads to NF-kB activation and the production of pro-inflammatory cytokines TNFα and IL-6. **2.** The activation of TNFR1 and IFNAR1 signaling pathway results in ZBP1 and RIPK3/ASC/CASP8 activation leading to MLKL phosphorylation and necroptosis induction. **3.** This can also leads to Caspase-3 cleavage and apoptosis induction. **4.** Self-dsDNA or mtDNA sensing by NLRP3 or AIM2 induces inflammsome formation leading to Gasdermin D cleavage enabling Gasdermin D pore formation and the release mature IL-1β and pyroptosis. NLRP3 inflammasome formation can be enhanced by the ZBP1/RIPK3/CASP8 complex. **5.** A second signal of STING activation with diABZI induces cell death and the release of self-DNA which is sensed by cGAS and form 2′3′-cGAMP leading to STING hyper activation, the amplification of TBK1/IRF3 and NF-kB pathway and the subsequent production of IFN-I and inflammatory TNFα and IL-6. This also leads to IFI204 and DDX41 upregulation thus, amplifying the inflammatory loop. The upregulation of apoptosis, pyroptosis and necroptosis is indicative of STING-dependent PANoptosis.

STING agonist diABZI induces neutrophilic lung inflammation and PANoptosis **A**, Airway STING priming induce a neutrophilic lung inflammation with epithelial barrier damage, double-stranded DNA release in the bronchoalvelolar space, cell death, NETosis and type I interferon release. **B**, **1.** The diamidobenzimidazole (diABZI), a STING agonist is internalized into the cytoplasm through unknown receptor and induce the activation and dimerization of STING followed by TBK1/IRF3 phosporylation leading to type I IFN response. STING activation also leads to NF-kB activation and the production of pro-inflammatory cytokines TNFα and IL-6. **2.** The activation of TNFR1 and IFNAR1 signaling pathway results in ZBP1 and RIPK3/ASC/CASP8 activation leading to MLKL phosphorylation and necroptosis induction. **3.** This can also leads to Caspase-3 cleavage and apoptosis induction. **4.** Self-dsDNA or mtDNA sensing by NLRP3 or AIM2 induces inflammsome formation leading to Gasdermin D cleavage enabling Gasdermin D pore formation and the release mature IL-1β and pyroptosis. NLRP3 inflammasome formation can be enhanced by the ZBP1/RIPK3/CASP8 complex. **5.** A second signal of STING activation with diABZI induces cell death and the release of self-DNA which is sensed by cGAS and form 2′3′-cGAMP leading to STING hyper activation, the amplification of TBK1/IRF3 and NF-kB pathway and the subsequent production of IFN-I and inflammatory TNFα and IL-6. This also leads to IFI204 and DDX41 upregulation thus, amplifying the inflammatory loop. The upregulation of apoptosis, pyroptosis and necroptosis is indicative of STING-dependent PANoptosis.

## Introduction

Stimulator of interferon genes (STING) is recognized as crucial in host immune responses against tumors [1], as the absence of STING leads to defective CD8^+^ T cell priming [2]. Cyclic dinucleotide (CDN) and non-nucleotidyl STING agonists are in development for cancer immunotherapy [1, 3]. Interesting activities in inhibiting infection by SARS-COV2, the agent responsible for COVID-19, in vitro and in vivo in mice have recently been reported [4, 5]. However, the clinical development of CDN STING agonists is hampered by limited bioavailability and adverse effects. In cancer therapy, subcutaneous or intratumor routes of administration are most frequent [3] while intravenous administration may lead to systemic inflammation with release type I IFN and cytokines in the bloodstream [6]. This triggered the development of encapsulated nanoparticulate forms of STING agonists [7], such as lysosomes loaded with cGAMP for airway delivery in the treatment of lung metastasis [8].

We showed the critical role of the STING pathway in sterile lung inflammation [9] after airway exposure to silica microparticles [10] or cigarette smoke [11], involving lung cell damage, cell death, and self-dsDNA release in the bronchoalveolar space that triggers STING activation and downstream type I IFN responses. We hypothesized that STING agonists administered in the airways for anticancer or anti-COVID treatment might induce an inflammatory response, fueled by STING-mediated cell death [12], and we questioned whether STING agonists induce acute respiratory distress syndrome (ARDS), through programmed cell death PANoptosis, including pyroptosis, apoptosis, necroptosis pathways [13].

Here we addressed the lung inflammatory response induced by endotracheal administration of an endogenous CDN cGAMP, STING agonist, and a non-nucleotide-based synthetic STING agonist comprising two linked, symmetry-related amidobenzimidazole-based compounds (diABZI) selected for its enhanced binding to STING and higher potency [14]. We show that airway application of STING agonists triggered a neutrophilic response in the bronchoalveolar space, induced cell death with the loss of epithelial barrier function and release of self-dsDNA, neutrophil extracellular traps (NETs), and lung inflammation. We hypothesized and showed that self-dsDNA release was accompanied by upregulation of DNA sensors such as cyclic GMP-AMP synthase (cGAS), IFN-γ-inducible protein 16 (IFI16) mouse orthologue IFI204, or DEAD-box helicase 41 (DDX41) that trigger type 1 IFN response through STING, TANK-binding kinase 1 (TBK1) and IFN regulatory factor 3 (IRF3) activation, as well as upregulation of Toll-like receptor 9 (TLR9) and the inflammasomes NLRP3 and AIM2. Markers of apoptosis, pyroptosis, and necroptosis were also present and colocalized in airway recruited inflammatory cells, indicative of STING-dependent PANoptosis.

## Results

### cGAMP local administration triggers airway neutrophilic inflammation

**Fig. 1:**
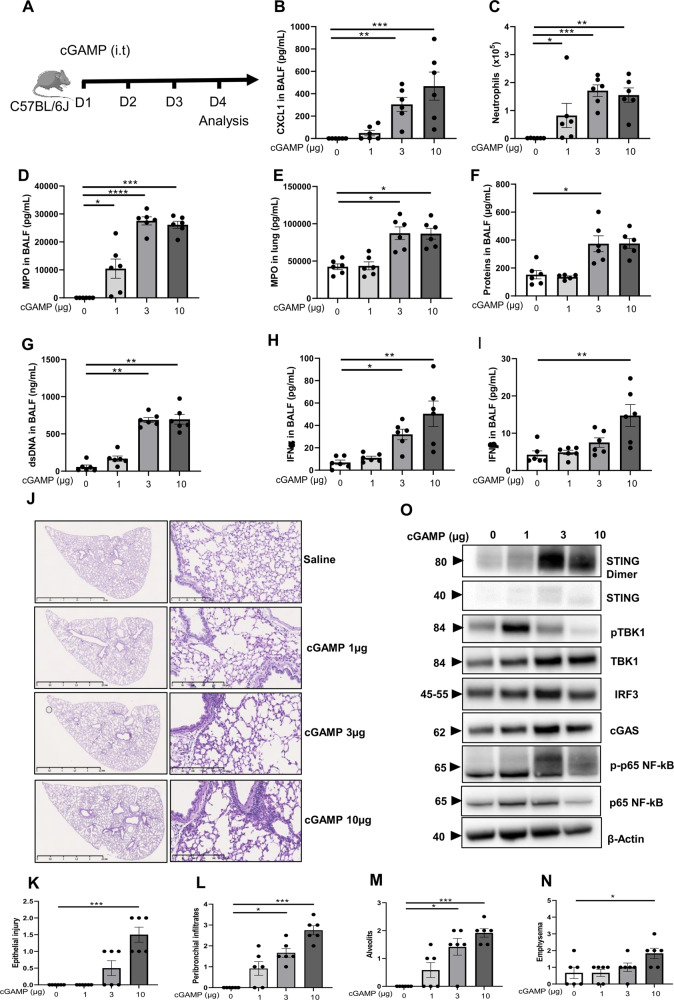
Endotracheal cGAMP induces neutrophilic inflammation, protein extravasation, and self-dsDNA release in the airways. **A** cGAMP (1, 3, or 10 µg, i.t.) or saline were administered daily in WT mice for 3 consecutive days, and parameters analyzed on day 4. **B** Concentration of CXCL1/KC in bronchoalveolar lavage fluid (BALF) determined by ELISA. **C** Neutrophils counts in BAL. **D**, **E** Myeloperoxidase (MPO) concentration in BALF (**D**) and in the lung (**E**) determined by ELISA. **F** Concentration of proteins in BALF. **G** Concentration of extracellular dsDNA in the acellular fraction of BALF. **H**, **I**. Concentration of IFNα and IFNβ in BALF determined by Luminex immunoassay. **J**–**N**. Lung tissue histology PAS staining (**J**), with pathology scoring of the presence of epithelial injury (**K**), peribronchial infiltration of inflammatory cells (**L**), alveolitis (**M**), and emphysema (**N**). Bars, left panels: 2.5 mm, right panels: 250 µm. **O** Immunoblots of STING pathway activation in the lung tissue in response to cGAMP, including STING dimer, STING, phospho-TBK1, TBK1, IRF3, cGAS, phosphorylated NF-κB p65 (p-p65- NF-κB), and p65 NF-κB, with β-actin as a reference. Graph data were presented as mean ± SEM with *n* = 6 mice per group. Each point represents an individual mouse. *p < 0.05, **p < 0.01, ***p < 0.001, ****p < 0.0001 (Nonparametric Kruskal–Wallis with Dunn post test).

We first addressed the effect of the natural cyclic dinucleotide STING ligand cGAMP in the airways. Endotracheal administration of cGAMP (1, 3, or 10 µg) for 3 consecutive days induced airway inflammation within 24 h (Fig. 1A). The neutrophilic attracting chemokine CXCL1/KC was released in the bronchoalveolar lavage fluid (BALF; Fig. 1B), and this was accompanied by increased neutrophil recruitment (Fig. 1C) and myeloperoxidase (MPO), a marker of neutrophil presence and activation in the BALF (Fig. 1D) and in the lung tissue (Fig. 1E). cGAMP-induced airway dysfunction was evidenced by increased total protein extravasation (Fig. 1F), self-dsDNA release in the bronchoalveolar space (Fig. 1G), and IFNα and IFNβ in the airways (Fig. 1H, I).

Thus, the natural CDN STING ligand cGAMP is a potent inducer of neutrophilic inflammation in the airways that may cause cell damage and lung dysfunction. Indeed, airway cGAMP resulted in acute alveolitis with thickened alveolar septae containing inflammatory cells, partial disruption of the alveolar membrane and rarefaction of alveoli, acute bronchiolitis with cell death and a beginning of remodeling with peribronchial infiltrates, visible 24 h after 1 µg, and further increased after 3-10 µg cGAMP endotracheal administration (Fig. 1J–N), but no mucus secretion (PAS staining). These are typical features following a massive injury with inflammation and incipient emphysema, repair, and fibrosis (Fig. 1J–N).

STING activation triggers several inflammatory pathways including IRF3 and NF-κB activation. We show that cGAMP administration at 1 to 10 µg increased STING dimers, TBK1 phosphorylation (pTBK1), IRF3, cGAS, NF-κB, and NF-κB p65 subunit phosphorylation (p-p65 NF-κB) in the lung tissue (Fig. 1O and Supplemental Fig. S5A). We next asked whether both pathways were functionally activated following the administration of cGAMP in the airways. Indeed, the chemokine *Cxcl10* mRNA, a typical Interferon-stimulated gene (ISG) downstream of IRF3/type I IFN, was overexpressed in the lung tissue after administration of 3-10 µg cGAMP in the airways (Supplemental Fig. S1A). CXCL10/IP-10 was increased in the BALF after 10 µg cGAMP administration (Supplemental Fig. S1B), as were TNFα and IL-6, both triggered in response to NF-κB pathway (Supplemental Fig. S1C, D). Interestingly, the expression of STING gene *Tmem173* doubled in the lung 1 day after cGAMP administration (Supplemental Fig. S1E).

Thus, airway cGAMP administration induces strong lung inflammation and injury, associated with dsDNA release, type I IFN and inflammatory cytokines in the lung, and overexpression of STING transcript.

### Amplification loop of STING pathway activation

Since airway cGAMP administration induced extracellular dsDNA release in the BALF, we hypothesized that this dsDNA might act as a danger signal, activate DNA sensors, and induce further type I IFN response.

**Fig. 2:**
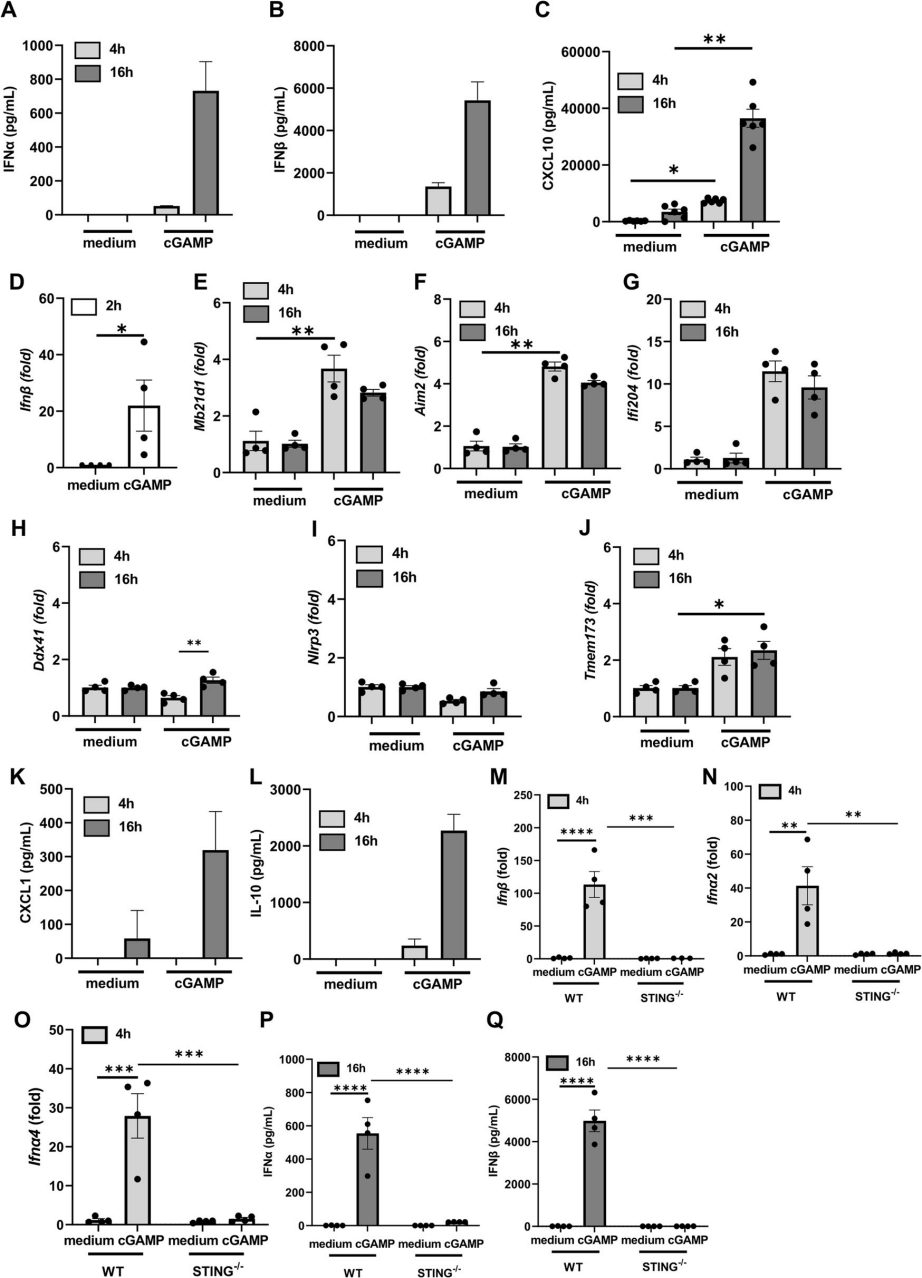
cGAMP-induced type I IFN macrophage response and DNA sensor expression. Bone marrow-derived macrophages from wild-type (**A**–**L**) and STING^−/−^ (**M**–**Q**) mice were unstimulated or stimulated with cGAMP (14 µM) for 4 or 16 h as indicated. **A**–**C** Protein concentrations of IFNα (**A**) and IFNβ (**B**) in macrophage culture supernatant determined by multiplex immunoassay, and of CXCL10 quantified by ELISA (**C**). **D**–**J** *Ifnb, Mb21d1, Aim2, Ifi204, Ddx41, Nlrp3*, and *Tmem173* transcripts measured by real-time PCR. **K**, **L** CXCL1 (**K**) and IL-10 (**L**) protein concentration quantified by ELISA. **M**–**O** *Ifnβ1, Ifnα2*, and *Ifnα4* transcripts measured by real-time PCR in macrophages from WT and STING^−/−^. **P**, **Q** Comparison of protein levels of IFNα (**P**) and IFNβ (**Q**), in the supernatant of WT and STING^−/−^ macrophages. Data were presented as mean ± SEM with *n* = 4 mice. *p < 0.05, **p < 0.01, ***p < 0.001, ****p < 0.0001 (**A**–**L** Nonparametric Kruskal–Wallis with Dunn post test. **M**–**Q** Two-way Anova with Tukey post test).

We analyzed the kinetics of cGAMP-induced responses in vitro in bone marrow-derived macrophages (Fig. 2). Type I IFNα and IFNβ release at 4 h, further increased 16 h after macrophage activation by cGAMP (Fig. 2A, B). CXCL10 was also strongly increased at 4–16 h (Fig. 2C). *Ifnβ* gene was already overexpressed 2 h after cGAMP addition (Fig. 2D), and phosphorylation of STING, TBK1 and IRF3 occurred already at 1 h, while STAT1 phosphorylation was delayed to 2 h (supplemental Fig S2 and S5G). ISG includes DNA sensors such as cGAS, AIM2, or IFI204, and we verified their expression. There was overexpression of cGAS gene *Mb21d1*, *Aim2*, and *Ifi204* genes at 4–16 h (Fig. 2E–G), while *Ddx41* and *Nlrp3* genes were barely affected (Fig. 2H, I). STING gene *Tmem173* itself was overexpressed at 16 h (Fig. 2J). CXCL1 and IL-10 were increased at 16 h (Fig. 2K, L). We verified that the effects of cGAMP was indeed mediated by STING using *Tmem173*-deficient (STING^−/−^) macrophages. As expected, *Ifnβ*, *Ifna2*, *and Ifna4* genes expression, and IFNα and IFNβ release were abrogated in STING^−/−^ macrophages (Fig. 2M–Q).

Thus, activation of STING by the natural CDN agonist cGAMP triggers a well-coordinated expression of type I IFNs and ISGs including several DNA sensors that may contribute to downstream chemokines and cytokines release.

### Synthetic STING agonist diABZI triggers potent inflammation in murine macrophages and human epithelial cells

**Fig. 3:**
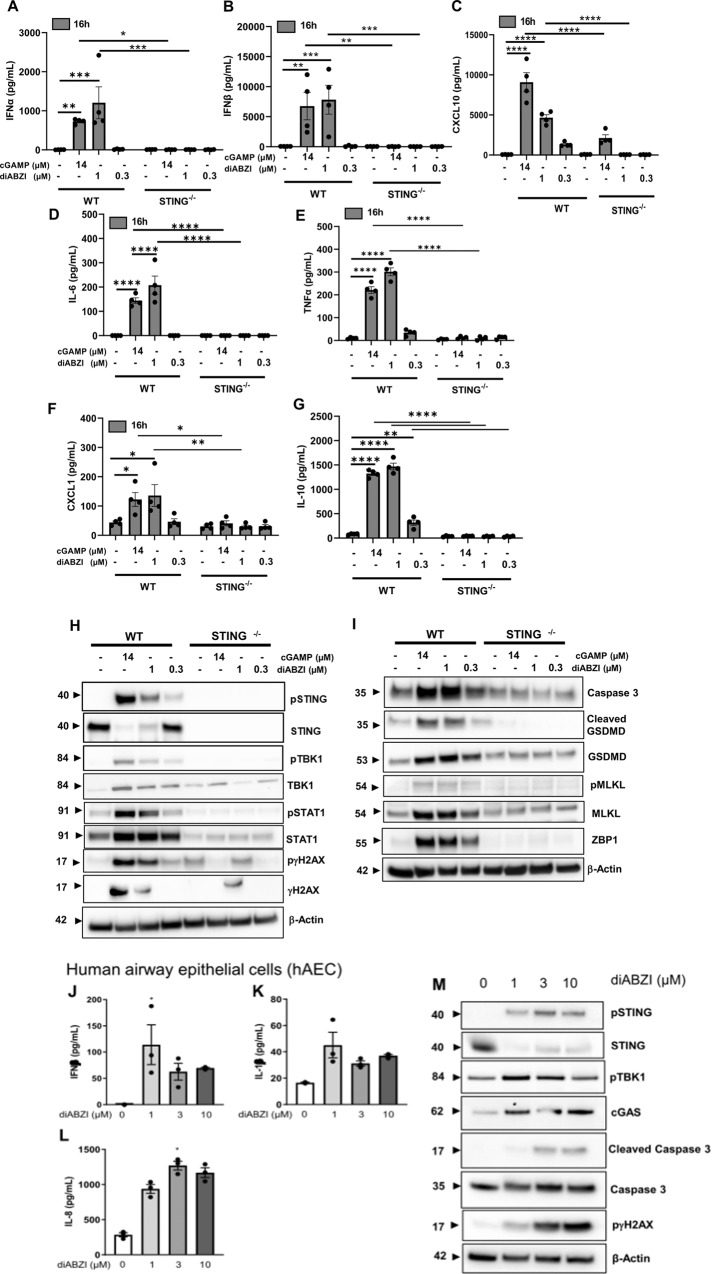
DiABZI-induced type I IFN response in human alveolar epithelial cells. **A**–**G** Bone marrow-derived macrophages (BMDM) from wild-type and STING^−/−^ mice were unstimulated or stimulated with cGAMP (14 µM) or diABZI (0.3 or 1 µM) for 16 h. Protein concentrations of IFNα (**A**) and IFNβ (**B**) in macrophage culture supernatant determined by multiplex immunoassay, of CXCL10 (**C**), IL-6 (**D**), TNFα (**E**), CXCL1 (**F**), and IL-10 (**G**) quantified by ELISA. **H**, **I** Immunoblots of STING axis (**H**), including phospho-STING, STING, phospho-TBK1, TBK1, phospho-STAT1, STAT1, DNA damage as revealed by phospho-γH2AX, γH2AX and of cell death axis (**I**) including Caspase 3, cleaved Gasdermin D, Gasdermin D, phospho-MLKL, MLKL, and ZBP1 in WT and STING ^−/−^ macrophages, with β-actin as a reference. **J**–**L** Human alveolar epithelial cells (hAEC) were unstimulated or stimulated with diABZI (1, 3, or 10 µM) for 24 h. Protein levels of IFNβ (**J**), IL-1β (**K**), and IL-8 (**L**) were released in the culture supernatant. **M** Immunoblots of STING axis, including phospho-STING, STING, phospho-TBK1, cGAS, and cell death markers cleaved Caspase 3, Caspase 3, and phospho-γH2AX with β-actin as a reference. *p < 0.05, **p < 0.01, ***p < 0.001, ****p < 0.0001, (**A**–**G**; two-way Anova with Tukey post test. **J**–**L**; nonparametric Kruskal–Wallis with Dunn post test). Data were presented as mean ± SEM and are representative of two independent experiments with *n* = 4 (**A**–**G**) and *n* = 3 (**J**–**L**) independent cultures.

To further characterize the effects of triggering STING activation in the airways, we turned to the recently developed and highly potent synthetic, non-nucleotidyl molecule diABZI [14]. We first checked the response induced by diABZI on murine macrophages. DiABZI at 1 µM induced the release of IFNα, IFNβ, CXCL10, IL-6, TNFα, CXCL1, and IL-10 at 16 h (Fig. 3A–G), similar to the natural CDN cGAMP, and this was abrogated in STING^−/−^ macrophages (Fig. 3A–G).

DiABZI at 0.3–1 µM induced the phosphorylation of STING and downstream TBK1 kinase, together with STAT1 phosphorylation, similar to cGAMP (Fig. 3H and supplemental Fig. S5B). The relative reduction of STING protein in conditions when STING is phosphorylated is intriguing, not due to antibody artifact as it is seen in independent blots, and might be related to the STING protein turnover in cells in culture in vitro. DNA damage was revealed by phosphorylated-γH2AX (pγH2AX; Fig. 3H and supplemental Fig. S4A). Indeed, cell death was also induced, as documented by immunoblots with elevated caspase 3, cleaved Gasdermin D (GSDMD) as a marker of pyroptosis, elevated Mixed Lineage Kinase domain Likepseudokinase (MLKL), and phospho-MLKL as a marker of necroptosis (Fig. 3I and supplemental Fig. S4A). This was suggestive of PANoptosis, and confirmed as the Z-DNA-binding protein 1 (ZBP1) was also upregulated (Fig. 3I and supplemental Fig. S5B). These responses were reduced in STING^−/−^ mice (Fig. 3H, I), documenting the STING dependence of the induced responses.

We next characterized the response induced by diABZI on primary human airway epithelial cells (hAEC). DiABZI at 1–10 µM induced the expression of IFNβ, IL-1β, and IL-8 (Fig. 3J–L). Indeed, diABZI induced the phosphorylation of STING and downstream TBK1 kinase, as well as overexpression of cGAS, but it also induced cell death, documented by cleaved Caspase 3 as a marker of apoptosis and DNA damage with increased pγH2AX (Fig. 3M and supplemental Fig. S5C).

Thus, diABZI induced activation of the STING pathway and cell death with upregulation of markers of apoptosis, pyroptosis, and necroptosis, indicative of STING-dependent PANoptosis, a term recently introduced [13].

### Airway administration of STING agonist diABZI triggers neutrophilic inflammation and NET formation

**Fig. 4:**
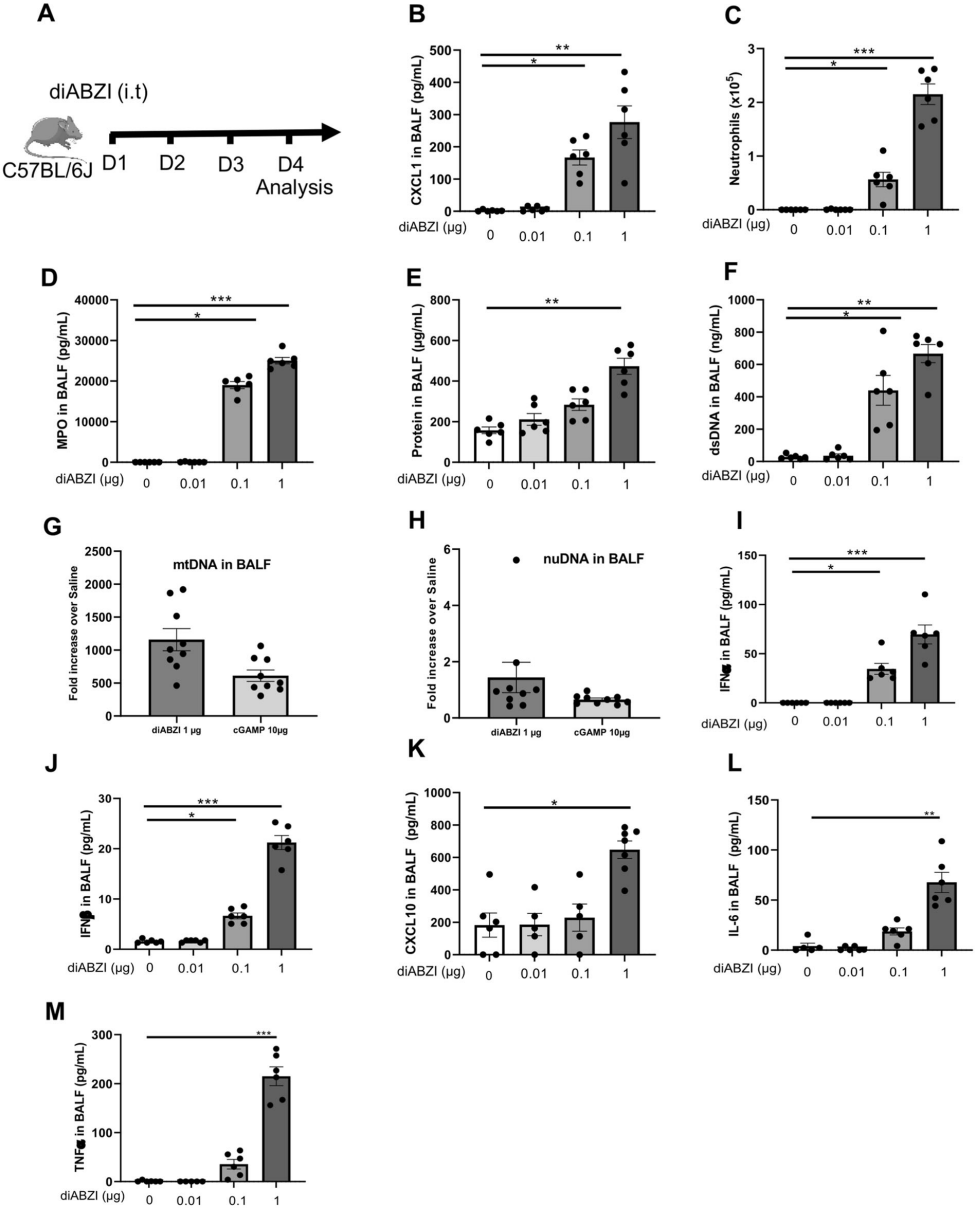

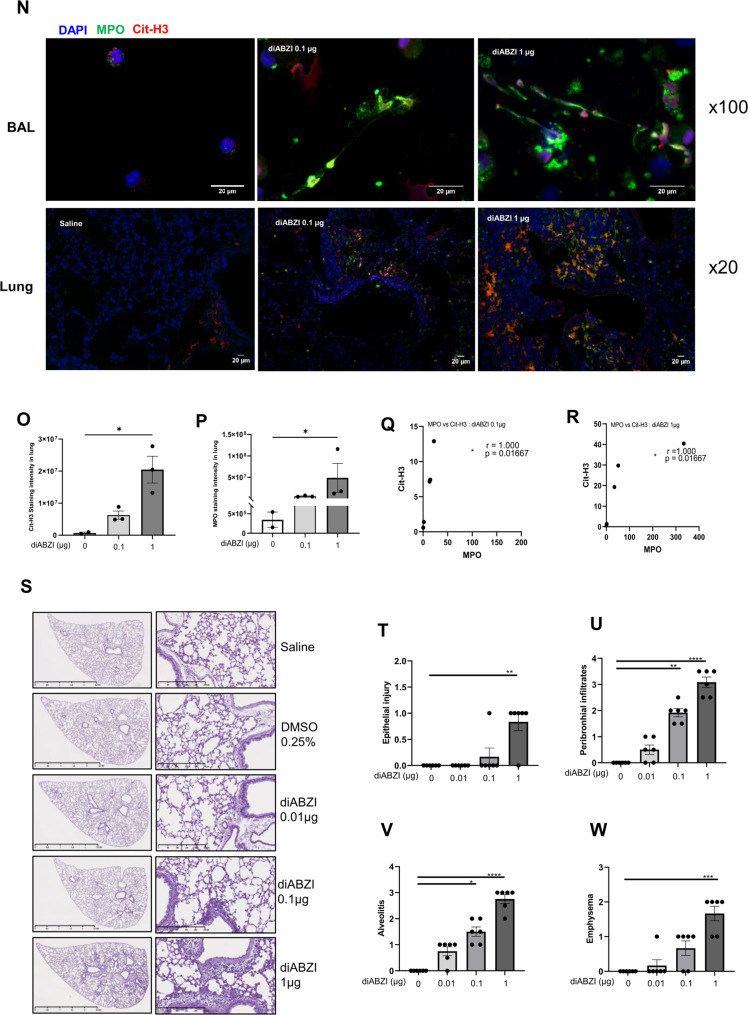
STING agonist diABZI triggers neutrophilic airway inflammation. **A** STING agonist diABZI (0.01, 0.1, or 1 µg, i.t.) DMSO (0.25%) vehicle or saline were administered daily in WT mice for 3 consecutive days and parameters were analyzed on day 4. **B** Concentration of CXCL1/KC in the bronchoalveolar lavage fluid (BALF) determined by ELISA. **C** Neutrophils counts in BAL. **D** Concentration of myeloperoxidase (MPO) in BALF determined by ELISA. **E** Concentration of proteins in BALF. **F** Concentration of extracellular dsDNA in the acellular fractions of BAL. **G**, **H** Mitochondrial DNA (mtDNA) (**G**) and nuclear DNA (nDNA) (**H**) in the BALF after diABZI or cGAMP administration. **I**–**M** IFNα (**I**) and IFNβ (**J**) determined by multiplex immunoassay, and CXCL10 (**K**), IL-6 (**L**), and TNFα (**M**) quantified by ELISA in the BALF. **N** Visualization of NETs in BAL and lung with the staining of DNA dye DAPI (cyan), MPO (green), and citrullinated Histone 3 (red). Bars, 20 µm. **O**, **P** Quantification of Cit-H3 staining intensity (**O**) and MPO staining intensity (**P**) in the lung. **Q**, **R** Correlation between MPO and Cit-H3 after diABZI administration at 0.1 µg (**Q**) and 1 µg (**R**). **S**–**W** Lung tissue histology PAS staining (**S**), with pathology scoring of the presence of epithelial injury (**T**), peribronchial infiltration of inflammatory cells (**U**), alveolitis (**V**), and emphysema (**W**). Bars, left panels: 2.5 mm, right panels: 250 µm. Graph data were presented as mean ± SEM with *n* = 5–6 mice per group. Each point represents an individual mouse. *p < 0.05, **p < 0.01, ***p < 0.001, ****p < 0.0001 (Nonparametric Kruskal–Wallis with Dunn post test).

Endotracheal administration of diABZI for 3 consecutive days induced a strong airway inflammation within 24 h (Fig. 4A). CXCL1 was released in the bronchoalveolar space after 0.1–1 µg diABZI administration (Fig. 4B), alongside neutrophil recruitment (Fig. 4C) and increased MPO (Fig. 4D). Protein extravasation in the bronchoalveolar space (Fig. 4E), indicative of disruption of the respiratory barrier, was accompanied by self-dsDNA release (Fig. 4F), suggesting cell damage and/or cell death. As cell stress or death may release mitochondrial DNA in the cytosol or extracellularly, that acts as a strong danger signal recognized by nucleic acid sensors such as cGAS, we quantified nuclear and mitochondrial DNA released in the BALF in response to diABZI or cGAMP. While nuclear DNA was barely released, there was a strong increase in mtDNA after diABZI 1 µg and to a lesser extent after exposure to cGAMP 10 µg (Fig. 4G, H), with a 35- to 42-fold mtDNA/nDNA ratio after cGAMP and diABZI exposure, respectively.

We next asked whether both IRF3 and NF-κB pathways were activated following STING activation by diABZI administration. Although no phosphorylated TBK1, IRF3, or p65 NF-κB could be detected at this time point (not shown), there was a functional activation of these pathways. Indeed, IFNα and IFNβ were released in the BALF after administration of diABZI at 0.1–1 µg (Fig. 4I, J), as was CXCL10, downstream of type I IFN/IRF3, after 1 µg diABZI (Fig. 4K), and both IL-6 and TNFα, triggered in response of NF-κB pathway (Fig. 4L, M).

DiABZI activating neutrophilic responses, we further investigated the formation of NETs, a neutrophil innate effector mechanism. Indeed, NETs comprising neutrophil-derived DNA, citrullinated histone H3 (Cit-H3), and MPO were increased by diABZI administration in the airways (Fig. 4N–R).

Histologically, marked inflammatory cell infiltration in the lung parenchyma was visible 24 h after three doses of 0.01 µg, and furthermore 0.1–1 µg diABZI endotracheal administration (Fig. 4S–W) with signs of epithelial injury, alveolitis, and emphysema, but no mucus secretion revealed by PAS staining.

Thus, the synthetic, non-nucleotidyl, STING ligand diABZI is a potent inducer of neutrophilic inflammation in the airways that may cause cell damage, dsDNA release and NETosis, and lung dysfunction.

### DiABZI-induced pulmonary cell death by PANoptosis

**Fig. 5:**
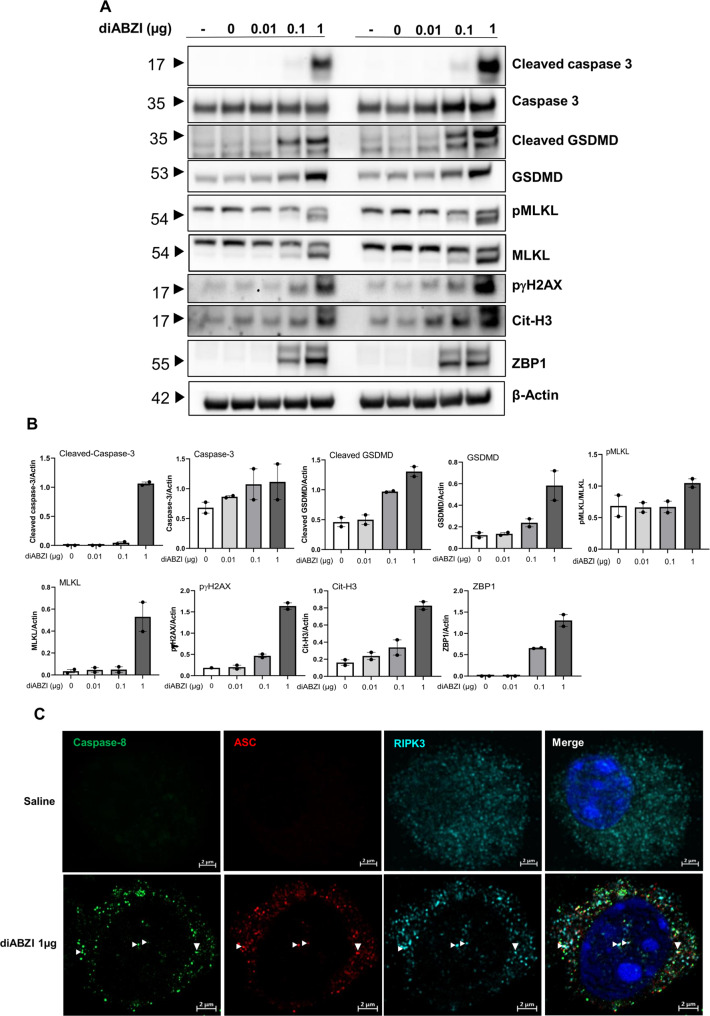

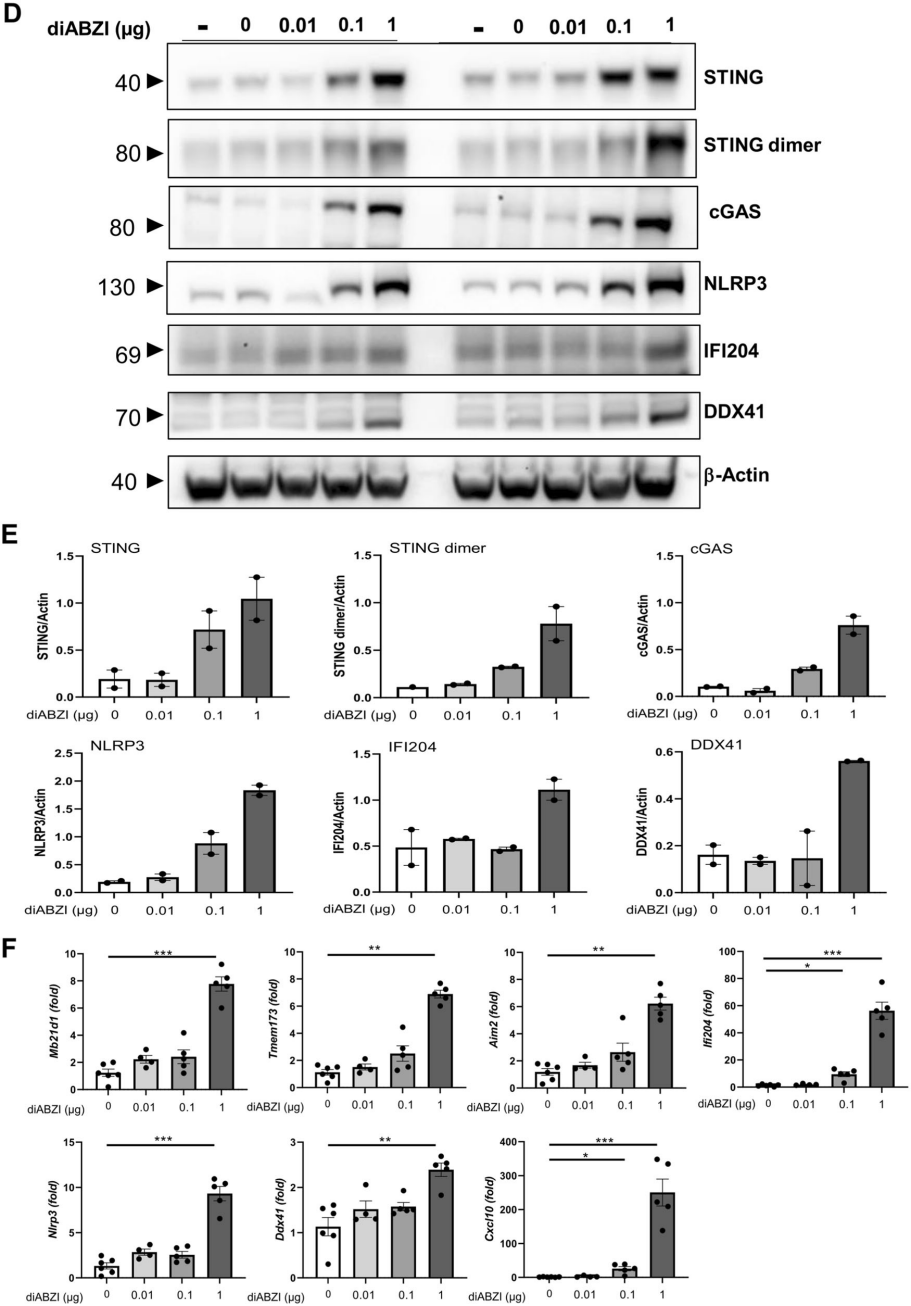
Airway diABZI induces lung tissue PANoptosis and damage and STING pathway activation. diABZI (0.01, 0.1, or 1 µg, i.t.) or saline were administered daily in WT mice for 3 consecutive days as in Fig. 4 and parameters analyzed on day 4. **A** Immunoblots of cleaved Caspase 3, Caspase 3, cleaved Gasdermin D, Gasdermin D, phospho-MLKL, MLKL, phospho-γH2AX, citrullinated Histone 3 (Cit-H3), and ZBP1 with β-actin as a reference. **B** Immunoblot quantification of cleaved Caspase 3, Caspase 3, cleaved Gasdermin D, Gasdermin D, pMLKL, MLKL, phospho-γH2AX, citrullinated Histone 3 (Cit-H3), and ZBP1, normalized to b-actin. **C** Confocal microscopy showing Caspase 8 (green), ASC (red), RIPK3 (far-red/turquoise blue), and DNA dye DAPI (cyan) in BAL cells of mice exposed to saline or diABZI 1 µg, showing the colocalization of PANoptosome components. Bars, 2 µm. **D** Immunoblots of STING axis in the lung of WT mice including STING and STING dimer, cGAS, NLRP3, IFI204, DDX41 with b-actin as reference. **E** Immunoblot quantification of STING and STING dimer, cGAS, NLRP3, IFI204, and DDX41 normalized to β-actin. **F** *Mb21d1, Tmem173, Aim2, Ifi204, Nlrp3, Ddx41*, *and Cxcl10* transcripts were measured by real-time PCR. *p < 0.05, **p < 0.01, ***p < 0.001. (Nonparametric Kruskal–Wallis test followed by Dunn post test). Graph data from real-time PCR are presented as mean ± SEM with *n* = 4–6 mice per group. Immunoblots representative of *n* = 2 samples from two independent experiments, quantified in bar graphs with *n* = 2.

As STING may mediate apoptosis [12, 15], we characterized the mode of cell death induced by diABZI in the lung. Airway administration of diABZI at 0.1 and more at 1 µg induced caspase 3 cleavage in the lung tissue, indicative of STING activation-induced apoptosis (Fig. 5A and supplemental Fig S5D). The occurrence of pyroptosis and necroptosis in diABZI-induced pulmonary cell death was assessed by immunoblots of cleaved Gasdermin D (GSDMD) and phosphorylated MLKL. Indeed, GSDMD and MLKL were increased in the lung, with the presence of cleaved GSDMD and phosphorylated MLKL (54 kD lower band) after administration of diABZI at 0.1 and further augmented at 1 µg (Fig. 5A, B). These data were suggestive of PANoptosis induced in the lung after exposition to diABZI, and was confirmed as the Z-DNA-binding protein 1 (ZBP1) was also upregulated (Fig. 5A, B). DNA damage was documented as increased levels of pγH2AX after diABZI treatment (Fig. 5A, B). Cit-H3 detection provided evidence of pulmonary NETosis triggered by diABZI administration (Fig. 5A, B).

To further characterize PANoptosis we next analyzed ASC-Caspase 8-RIPK3 complex formation [16, 17]. Indeed, in sterile inflammation, the Caspase 8-RIPK3 shared by apoptosis and necroptosis is generally associated with ASC, the adapter protein of inflammasomes [18], these molecular interactions likely serving as the backbone for PANoptosome formation. Confocal microscopy analysis of immunofluorescence labeling shows increased Caspase 8, ASC, and RIPK3 protein expression in BAL cells of mice exposed to diABZI 1 µg as compared to saline controls, and colocalization of these PANoptosome components, indicative of PANoptopsis complex (Fig. 5C).

Therefore, triggering airway STING activation by local administration of a potent STING agonist, diABZI, induced lung cell death through apoptosis, necroptosis, and pyroptosis, with evidence of PANoptosis complex formation, indicating STING-induced PANoptosis.

### DiABZI-induced nucleic acid sensors and inflammasome expression

We next asked whether triggering the STING pathway induced further DNA sensors expression. We report STING overexpression and activation visible by increased STING dimers in immunoblots of lung tissue after endotracheal administration of 0.1–1 µg diABZI (Fig. 5D, E and supplemental Fig S5E). cGAS protein expression was also strongly increased in the lung after the diABZI challenge (Fig. 5D, E). DNA sensors cGAS gene *Mb21d1* and AIM2 are ISG, and their messages increased after the diABZI challenge, as were *Tmem173*, *Ifi204*, *Nlrp3*, less *Ddx41*, and >200-fold *CxCl10* (Fig. 5F).

Therefore, triggering airway STING activation by local administration of the potent STING agonist diABZI induced DNA damage and overexpression of DNA sensors and inflammasomes that may fuel further inflammatory responses.

### Self-dsDNA release contributes to diABZI-induced airway inflammation

**Fig. 6:**
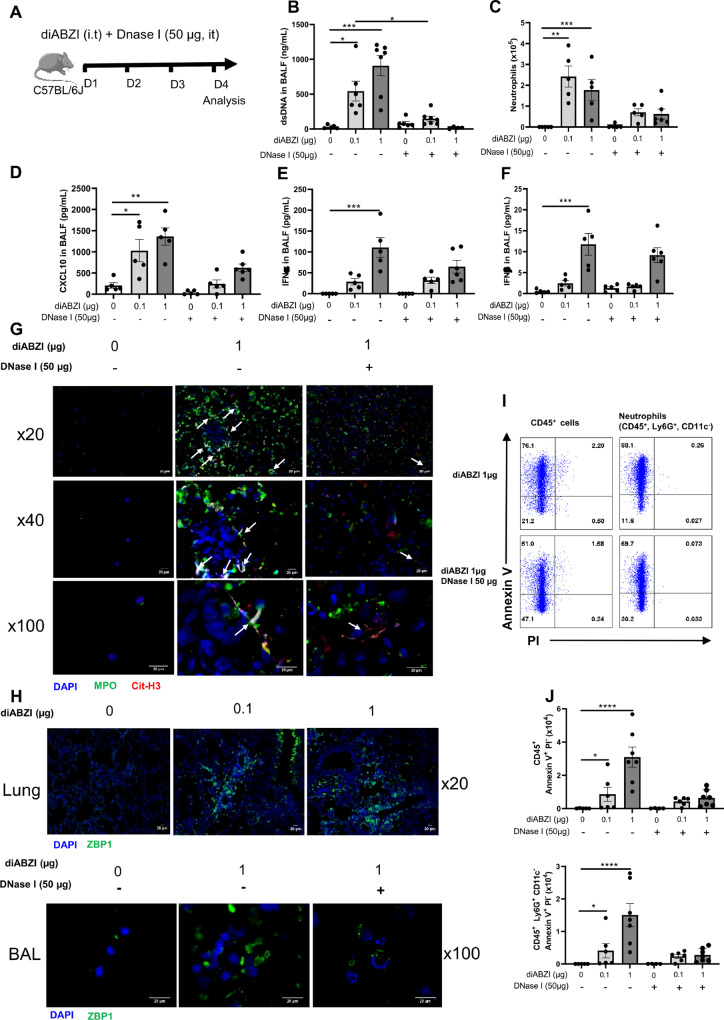

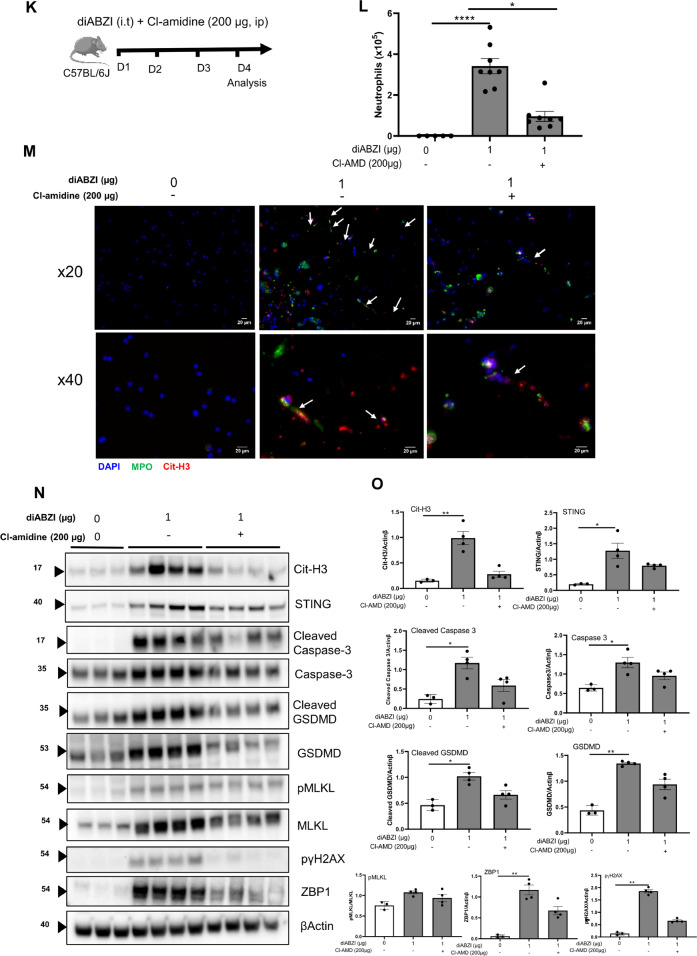

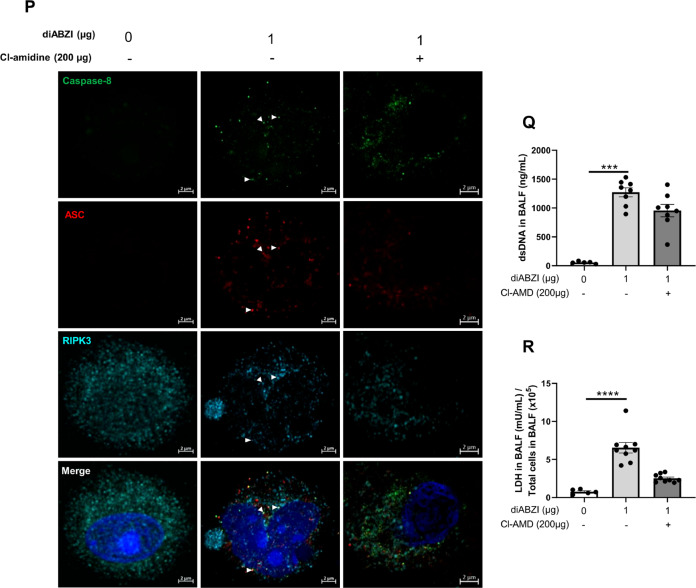
DNase I treatment or NET inhibition reduce diABZI-induced neutrophilic airway inflammation. **A** diABZI (0.1 or 1 µg, i.t.) were administered with DNase I (50 µg/mouse, i.t.) daily in WT mice for 3 consecutive days and parameters were analyzed on day 4. **B** Extracellular dsDNA in acellular fraction of BAL. **C** Neutrophils counts in BAL. **D** Concentration of CXCL10 in BALF, determined by ELISA. **E**, **F** IFNα (**E**) and IFNβ (**F**) in BALF are determined by multiplex immunoassay. **G** Visualization of NETs with staining of DNA (DAPI, cyan), MPO (green), and Cit-H3 (red) in cells from BAL. Bars 20 µm, magnification x20 upper panels, x40 middle panels, x100 lower panels. **H** Visualization of PANoptosis with stainings of ZBP1 (vert) and DNA (DAPI, cyan) in cells from lung and BAL. Bars 20 µm, magnification x20 (Lung), x100 (BAL). **I**, **J** Annexin V/PI flow cytometry analysis pre-gated on singlet cells, and CD45^+^ (leukocytes) or CD45^+^Ly6G^+^CD11C^−^ (neutrophils). **K** diABZI (1 µg, i.t.) was administered alone or with Cl-amidine (200 µg/mouse, i.p.) daily in WT mice for 3 consecutive days and parameters were analyzed on day 4. **L** Neutrophils counts in BAL. **M** Visualization of NETs with staining of DNA (DAPI, cyan), MPO (green), and Cit-H3 (red) in cells from BAL; Bars 20 µm, magnification x20 upper panels, x40 lower panels. **N** Immunoblots of Cit-H3, STING, cleaved caspase 3, caspase 3, cleaved Gasdermin D, Gasdermin D, phospho-MLKL, MLKL, phospho-γH2AX, and ZBP1 in the lung of WT mice exposed to diABZI alone or treated with Cl-amidine, with β-actin as reference. **O** Immunoblot quantification of Cit-H3, STING, cleaved caspase 3, caspase 3, cleaved GSDMD, GSDMD, phospho-γH2AX and ZBP1 normalized to β-actin, and phospho-MLKL normalized to MLKL. **P** Confocal microscopy showing Caspase 8 (green), ASC (red), RIPK3 (far-red/turquoise blue), and DNA dye DAPI (cyan) in BAL from mice challenged with diABZI at 1 µg and treated with Cl-amidine or saline. PANoptosome formation is illustrated by colocalization of the components of PANoptosome in the merged image (indicated by arrowheads). Bars, 2 µm. **Q** Concentration of extracellular dsDNA in the acellular fraction of BALF. **R** Lactate dehydrogenase (LDH) quantification in the acellular fraction of BALF. Graph data were presented as mean ± SEM with *n* = 5–8 mice/group. Each point represents an individual mouse. *p < 0.05, **p < 0.01, ***p < 0.001. (Nonparametric Kruskal–Wallis test followed by Dunn post test).

To evaluate the contribution of the self-dsDNA released after diABZI administration to the induced lung inflammation through secondary responses, we treated mice receiving local airway diABZI with DNase I (Fig. 6A). Pulmozyme DNase I treatment at 50 µg i.t. on the 3 consecutive days of diABZI administration efficiently abrogated dsDNA in the BALF (Fig. 6B), and reduced neutrophil recruitment and CXCL10 release in the airways (Fig. 6C, D), while other parameters such as IFNα, IFNβ (Fig. 6E, F), MPO, IL-6, or TNFα were barely affected (Supplemental Fig. S3A–C).

DNase I treatment resulted in the impairment of NETs formation as evidenced by DNA, MPO and citrullinated Histone 3 immunofluorescence analysis (Fig. 6G), and decreased ZBP1 protein in the airways (Fig. 6H).

The CD45^+^ inflammatory cells recruited in the airways after diABZI local administration showed increased frequency and absolute counts of necrotic Annexin V^+^ Propidium Iodide (PI)^+^ and apoptotic Annexin V^+^ PI^−^ cells, that were largely abrogated by the DNase I treatment (Fig. 6I, J and supplemental Fig. S3D).

To evaluate the interplay between STING activation and NETs formation, we treated mice receiving local airway diABZI with Cl-amidine, an inhibitor of peptidyl arginine deiminase 4 (PAD4), an enzyme essential for NET formation (Fig. 6K). Cl-amidine treatment at 200 µg i.p. on the 3 consecutive days of diABZI administration efficiently prevented neutrophil recruitment and NETs in the BALF (Fig. 6L, M). Treatment with Cl-amidine also reduced NETs formation as evidenced by Cit-H3 immunoblot, and the expression of STING protein in the lung (Fig. 6N, O).

To determine the link between NETosis and PANoptosis, we evaluated PANoptosis markers following Cl-amidine treatment in diABZI exposed mice. Cl-amidine treatment reduced diABZI-induced PANoptosis as evidenced by reduced cleaved caspase 3, caspase 3, cleaved GSDMD, GSDMD, pMLKL, MLKL, and ZBP1 proteins in lung, assessed by western blot (Fig. 6N, O and supplemental Fig S5F). Caspase 8, ASC, and RIPK3 protein expression in BAL cells was also reduced after Cl-amidine treatment, as seen by immunofluorescence (Fig. 6P). The diABZI-induced release of dsDNA in the BALF (Fig. 6Q), damaged DNA documented by immunoblot of pγH2AX in the lung (Fig. 6N, O), and overall lytic cell death measured by Lactate dehydrogenase (LDH) release in the BALF (Fig. 6R) were alleviated by Cl-amidine treatment.

Thus, the airway inflammatory response to diABZI local administration is in part dependent on the released dsDNA and NET formation.

### DNA sensors dependence of diABZI-induced airway inflammation

**Fig. 7:**
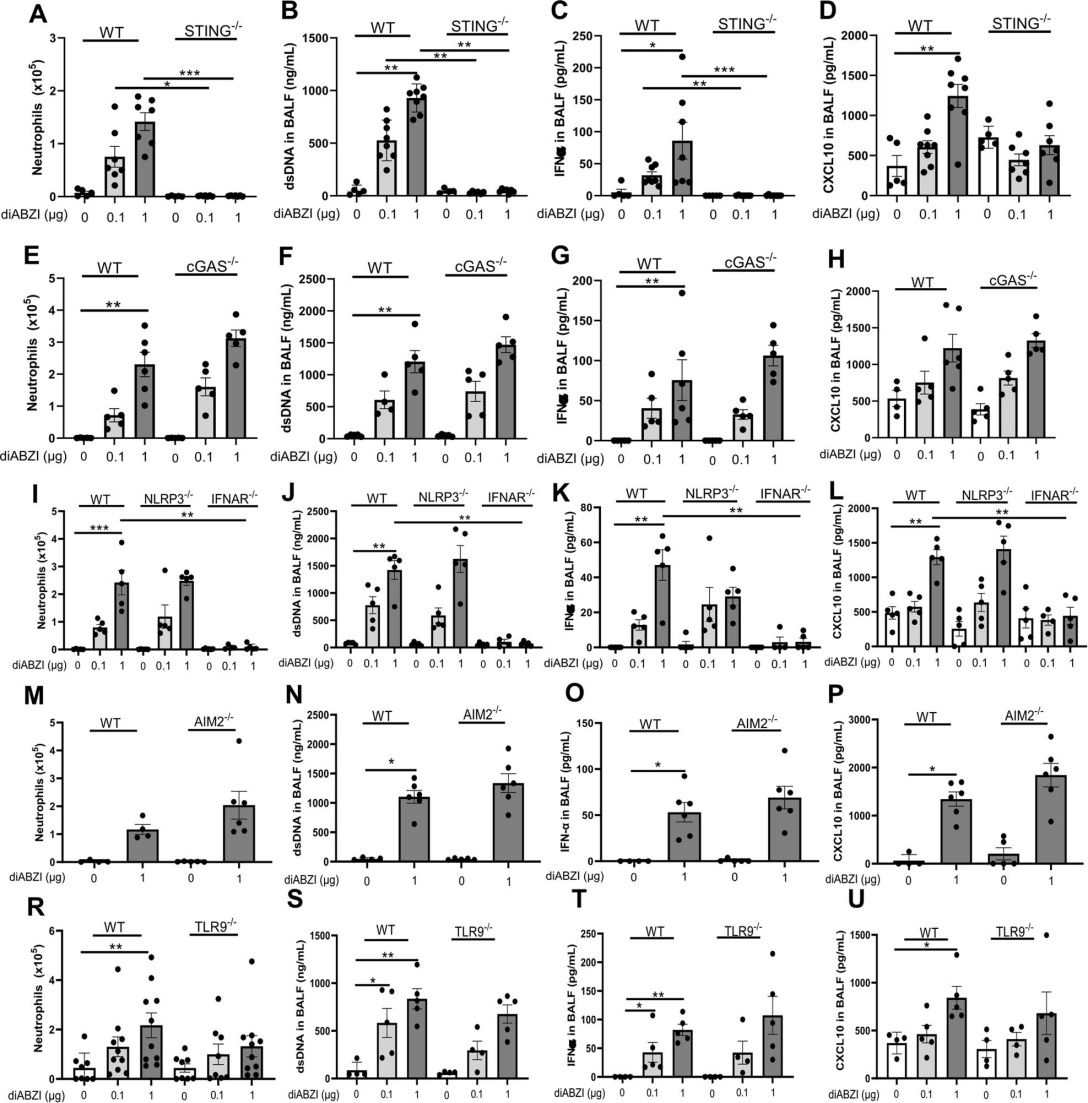
STING is the major sensor of diABZI-induced lung inflammation. DiABZI (0.1 or 1 µg, i.t.) or saline were administered daily in STING^−/−^, cGAS^−/−^, NLRP3^−/−^, IFNAR^−/−^, AIM2^−/−^, TLR9^−/−^, and WT mice for 3 consecutive days and parameters analyzed on day 4. **A–D** Neutrophils (**A**), concentrations of dsDNA (**B**), IFNα (**C**), and CXCL10 (**D**) in BALF in WT and STING^−/−^ mice. **E–H**. Neutrophils (**E**), dsDNA (**F**), IFNα (**G**), and CXCL10 (**H**) concentration in BALF in WT and cGAS^−/−^ mice. **I**–**L** Neutrophils (**I**), dsDNA (**J**), IFNα (**K**), and CXCL10 (**L**) concentration in BALF in WT and NLRP3^−/−^ and IFNAR^−/−^ mice. **M–P** Neutrophils (**M**), dsDNA (**N**), IFNα (**O**), and CXCL10 (**P**) concentration in BALF in WT and AIM2^−/−^ mice. **R–U** Neutrophils (**R**), dsDNA (**S**), IFNα (**T**), and CXCL10 (**U**) concentration in BALF in WT and TLR9^−/−^ mice. Graph data were presented as mean ± SEM with *n* = 4–10 mice/group, each point representing an individual mouse. *p < 0.05, **p < 0.01, ***p < 0.001. (Nonparametric Kruskal–Wallis test followed by Dunn post test).

As STING agonist cGAMP may activate alternative, noncanonical inflammasome priming, we first verified that the inflammatory airway response to diABZI administration is dependent on the STING pathway (Fig. 7A–D). Indeed, the recruitment of neutrophils and dsDNA release in the airways seen after endotracheal administration of 0.1–1 µg diABZI for 3 consecutive days in wild-type mice was absent in STING^−/−^ mice (Fig. 7A, B), as well as MPO release in BALF (Supplemental Fig. S4A). Further, IFNα and IFNβ release in the airways was absent in STING^−/−^ mice (Fig. 7C and Supplemental Fig. S4B), as were both CXCL10, downstream of IRF3/type I IFN (Fig. 7D), and NFκB-dependent IL-6 and TNFα (Supplemental Fig. S4C, D).

As dsDNA was released and cGAS expression was increased after diABZI administration, we then assessed the contribution of self-dsDNA-induced secondary responses, in cGAS-deficient mice. Interestingly, the airway inflammation induced by local diABZI administration was retained in cGAS^−/−^ mice, in terms of neutrophil recruitment, MPO, dsDNA, or cytokine release in the airways, including IFNα and IFNβ, CXCL10, IL-6, or TNFα, (Fig. 7E–H and supplemental Fig. S4E–H). This indicated a cGAS-independent response to diABZI and downstream dsDNA released. Similarly, while NLRP3 and AIM2 expression was increased after diABZI administration, the response to airway diABZI administration was unaffected in NLRP3^−/−^ and AIM2^−/−^ mice, in terms of neutrophil recruitment, MPO, dsDNA, or cytokine release in the airways, including IFNα and IFNβ, CXCL10, IL-6, or TNFα (Fig. 7I–P and supplemental Fig. S4I–P). As expected, the inflammatory responses to diABZI were abrogated in IFNAR^−/−^ mice (Fig. 7I–L and supplemental Fig. S4I–L), and the neutrophilic response was reduced in TLR9^−/−^ mice (Fig. 7R–U and supplemental Fig. S4R–U).

Thus, the airway inflammatory response to diABZI local administration is fully dependent on STING and IFNAR, and extracellular DNA and TLR9 are central to diABZI-induced neutrophilic response, independently of cGAS, NLRP3, and AIM2 pathways.

## Discussion

Immunotherapy with STING agonists is promising in oncology including lung cancer, and in fighting SARS-COV2 airway infection in mice [4, 5]. As the STING pathway fuels sterile lung inflammation after exposure to silica microparticles [10] or cigarette smoke [11], we characterized the lung inflammatory response induced by endotracheal administration of STING agonists, endogenous CDN cGAMP, and synthetic diABZI. STING agonists triggered a neutrophilic response in the bronchoalveolar space, a strong pulmonary inflammatory response with loss of epithelial barrier function and ARDS, cell death and release of self-dsDNA, NETs formation, and type I IFN responses. Further, we show that STING agonists induce programmed cell death with features of pyroptosis, apoptosis, and necroptosis, indicative of STING-dependent PANoptosis and that extracellular dsDNA is central to diABZI-induced neutrophilic response.

We questioned the origin of dsDNA released after administration of STING agonists in the airways. cGAMP has noncanonical functions in inflammasome activation, enhancing the expression of inflammasome components such as NLRP3 through IFN-I-dependent response, while activating the inflammasomes through an AIM2, NLRP3, ASC, and caspase-1 dependent process [19]. STING activation may orchestrate a lysosomal cell death program engaging NLRP3 inflammasome after cytosolic DNA recognition [12]. The complex interactions of cGAS-STING signaling and different cell death pathways has been reviewed recently [20] and the role of STING in PANoptosis questioned. Here, we show that triggering STING directly via diABZI STING agonist administration triggered apoptosis, NETosis, and DNA damage, as evidenced by an increased presence of cleaved caspase 3, Cit-H3, and pγH2AX in the lung of treated mice. In addition, we show in the same setting that markers of pyroptosis, apoptosis, and necroptosis were present, and colocalized in inflammatory cells recruited in the airways, indicative of STING-induced PANoptosis.

The ectopic presence of nucleic acids in the cytosol represents a danger signal activating the cytosolic surveillance system, which may, in turn, activate DNA sensors. This is particularly the case for mitochondrial DNA, released after cellular and mitochondrial stress. Here, exposure to STING agonists yielded predominantly release of mitochondrial DNA over nuclear DNA in the airway. Self-dsDNA was an integral part of the response, as degradation of extracellular DNA by local administration of DNase I prevented diABZI-induced neutrophilic response in the airways. The expression of several DNA sensors, including cGAS, NLRP3, AIM2, IFI204, and DDX41, was increased after STING agonist-induced lung inflammation. While the airway inflammatory response to diABZI was fully dependent on STING and type I IFN, as expected, we showed that TLR9 is central to diABZI-induced neutrophilic response, independently of cGAS, NLRP3, and AIM2 pathways. Although CpG nucleotides are likely masked by methylation in mammalian self-DNA, cationic antimicrobial peptides such as LL37, human β-defensin (hBD)2, hBD3, or lysozyme condense self-DNA into particles that are endocytosed and trigger TLR9 activation and type I response in pDC and psoriatic skin lesions [21, 22].

In conclusion, the present findings establish that administration of STING agonists in the airways, besides activating type I IFN response, induces PANoptosis, dsDNA release, and NETosis, leading to neutrophilic lung inflammation and ARDS in a cGAS-independent, TLR9-dependent manner. Targeting STING activation as a therapeutic strategy for local cancer metastasis or infection may thus include a component of lung inflammation.

## Methods

### Mice

Female C57BL/6Rj mice were purchased from Janvier Laboratories (Le Genest St Isle, France). Wild-type mice and mice deficient for STING (STING^−/−^) [23], cGAS (cGAS^−/−^) [24], IFNAR (IFNAR^−/−^) [25], TLR9 (TLR9^−/−^) [26], NLRP3 (NLRP3^−/−^) [27], AIM2 (AIM2^−/−^) [28] were bred and housed under specific pathogen-free conditions at CNRS animal facility (TAAM UAR44, Orleans, France). They were maintained in a 12-h light-dark cycle with food and water ad libitum, following European and local legislation. Age-matched, 8- to 12- week-old mice were used for experiments. All animal experiments complied with the French Government animal experiment regulations and ARRIVE guidelines. The protocols were submitted to the “Ethics Committee for Animal Experimentation of CNRS Campus Orleans” (CCO) under numbers CLE CCO 2015-1087 and CLE CCO 2020-2018 and approved by the French Minister under APAFIS #19361 and #25360.

### In vivo animal experiments

Mice were anesthetized with 2% Isoflurane (ISO-VET, Netherlands) and challenged intratracheally (i.t) with cGAMP (1–10 µg in 40 µL, Invitrogen, California) or diABZI, a STING agonist 3 (0.01 to 1 µg in 40 µL, Cayman chemicals, Michigan) or saline (NaCl 0.9%) for 3 consecutive days. Bronchoalveolar lavage (BAL) was performed 24 h after the last challenge by flushing lung tissue four times with 0.5 mL of cold NaCl 0.9% via a cannula inserted in the trachea. BALF was collected, cells counted and cytospins performed. The supernatant of the first lavage was collected after centrifugation and stored at −80 °C for dsDNA quantification. The left lung lobe was harvested for histology, the post caval lung for RNA extraction and qPCR analysis, and the right lobes for Western blots analysis and cytokines measurement. Protein extravasation in the BALF was measured by Pierce™ BCA Protein Assay (ThermoFisher^®^, Massachusetts).

### Immunoblots

Lung tissues were homogenized in T-PER™ buffer supplemented with a cocktail containing protease and phosphatase inhibitors (halt™, ThermoFisher). Total protein was extracted and quantified by using Pierce™ BCA Protein Assay Kit (ThermoFisher®). Total protein (40 µg) was treated with NuPAGE™ LDS sample buffer and sample reducing agent (ThermoFisher®), and heated 10 min at 70 °C. Samples were resolved on 4–12% polyacrylamide gel (Bolt™ Mini protein gel, ThermoFisher) and run at 160 V for 45 min using the Mini gel Tank (ThermoFisher^®^). Total proteins were electroblotted to 0.2 µm nitrocellulose membrane (Amersham™, UK) using a Trans-Blot SD Transfer System (Bio-Rad, California) at 100 V for 45 min. Successful protein transfer was confirmed by using Ponceau S staining. Membranes were blocked with 5% nonfat milk (Cell signaling, Massachusetts) in 1X TBS-T (20 mM Tris Base, 150 mM sodium chloride, and 0.05% Tween-20 pH 7.6) for 1 h at room temperature.

Full membranes or portions of membranes were incubated overnight using primary antibodies from rabbit anti-phospho-STING (#72971 1/500; Cell signaling), anti-STING (#13647 1/500; Cell signaling), anti-phospho-TBK1 (#5483 1/500; Cell signaling), anti-TBK1 (#3504 1/500; Cell signaling), anti-phospho-IRF3 (#4947 1/500; Cell signaling), IRF3 (#4302 1/500; Cell signaling), anti-phospho-NF-kB (#3031 1/500; Cell signaling), anti- NF-kB (#4764 1/500; Cell signaling), anti- cGAS (#31659 1/500; Cell signaling), anti-NLRP3 (#15101 1/500; Cell signaling), anti-MLKL (#37705 1/500; Cell signaling), anti-phospho-MLKL (#37333 1/500; Cell signaling), anti-GSDMD (#ab209845 1/500; Abcam), anti-cleaved GSDMD (#10137 1/500; Cell signaling), anti-cleaved Caspase 3 (#9661 1/500), anti-Caspase 3 (#9662 1/500; Cell signaling), anti-phospho-γH2AX (#9718 1/500; Cell signaling), anti-γH2AX (#7631 1/500; Cell signaling), anti-histone H3 (#ab5103 1/500; Abcam, UK), anti-IFI204 (#ab228512 1/500; Abcam), anti-phospho-STAT1 (#9167 1/500; Cell signaling), anti-STAT1 (#14994 1/500; Cell signaling), mouse anti-ZBP1 (#SC-271483 1/300; Santa Cruz, Texas), and anti actin (#A3854 1/10000; Sigma-Aldrich, Massachusetts). Membranes were washed in TBS-T three times for 10 min each at room temperature, and then incubated with goat anti-rabbit-IgG-HRP-conjugate (#7074 1/2000; Cell signaling) or horse anti-mouse-IgG-HRP-conjugate (#7076 1/2000; Cell signaling) diluted in 5% nonfat milk in TBS-T for 1 h at RT.

The membranes were washed three times in TBS-T. Protein bands were visualized following exposure of the membrane to Amersham ECL™ prime substrate solution (Cytiva, Massachusetts) on film (PXi gel doc system^®^, India), and quantified by densitometry analysis using ImageJ software. Uncropped immunoblot gels are shown in Supplemental Fig. S5.

### Immunofluorescence staining of lung and BAL cells

Lungs were fixed with 4% formalin for 72 h, embedded in paraffin, and sectioned at 3 µm. Lung sections were dewaxed and rehydrated, then heated 20 min at 80 °C in citrate buffer 10 mM pH = 6 for antigen retrieval (unmasking step). Lung sections were permeabilized in PBS 0.5% triton X-100, blocked with 5% FCS for 1 h at RT, and then incubated overnight with primary goat antibodies to MPO (1:40, R&D systems, Minneapolis) and rabbit anti-H3-citrulline (1:100, Abcam, UK) or mouse anti- ZBP1 (1:100, Santa Cruz, Texas). After washing, the sections were incubated with donkey anti-rabbit IgG secondary antibodies conjugated with AlexaFluor568 (1:500, Invitrogen, Massachusetts) and anti-goat IgG secondary antibodies conjugated with AlexaFluor488 (1:200, Invitrogen) or with goat anti-mouse IgG conjugated with AlexaFluor488 in 1% FCS. Following washing, lung sections were stained with DAPI (1:1000) for 10 min, washed with PBS, and mounted onto microscope slides (Fluoromount-G, Invitrogen).

Cytospin slides were fixed in 4% PFA. Cells were washed three times in TBS, incubated 15 min in TBS-0.3% Triton X-100, then washed three times in TBS, blocked in TBS-10% FCS for 45 min and incubated overnight at 4 °C with primary antibodies to MPO (1:40, R&D systems), and to histone H3-citrulline (1:100, Abcam) for NETs visualization. For PANoptosome visualization slides were incubated with primary antibodies from mouse anti-ZBP1 (1:100, Santa Cruz), rat anti-Caspase 8 (1: 100, Enzo Lifesciences, France), goat anti-ASC (1:50, Abcam), and mouse anti-RIPK3 (B-2) Alexa Fluor^®^ 647 (1:30, Santa Cruz Biotechnology). After washing, slides were incubated as described above for NETs formation and ZBP1 detection. For analyse of PANoptosome formation, slides were incubated with donkey anti-goat IgG secondary antibodies conjugated with AlexaFluor488 (1:200, Invitrogen) and goat anti-rat secondary antibodies Alexa Fluor 546. Slides were stained using DAPI for 10 min. For NETs and ZBP1 visualization, cells were observed using a Leica DM 6000B microscope (Leica Camera, Wetzlar, Germany), images were acquired using MetaMorph^®^ software, and were treated using ImageJ software. For PANoptosme visualization, cells were observed using a Zeiss LSM 980 microscope coupled with a Zeiss Airyscan 2 device (Carl Zeiss Co. Ltd., Jena, Germany). Images were acquired using Zeiss LSM Image Browser (Carl Zeiss Co. Ltd., Jena, Germany).

### Flow cytometry

Bronchoalveolar lavage cells were counted and plated on a 96 well plate for extracellular staining. Different subsets of lung infiltrating cells or resident cells were detected by flow cytometry in BAL cell suspensions using a mix of the following fluorochrome-conjugated antibodies against mouse (CD45-PE-Cy7 1/200, eBiosciences, California), (Siglec F-PE-Cf59, 1/300, BD Bioscience, New Jersey), (Ly6G-FITC, 1/200), (CD3-AF700, 1/100, BD bioscience), (B220-BV711, 1/200), (Epcam-SB645, 1/100, Invitrogen, Massachusetts), (CD11c-BV605, 1/200), (F4/80-BV42, 1/200), and (Fc Block, 1/200) to avoid nonspecific binding. Early apoptosis to late apoptosis/necrosis in the detected cell populations was assessed by using Annexin V-APC and Propidium Iodide Apoptosis Detection Kit I (BD Bioscience). All staining reactions were performed at RT for 20 to 30 min. Flow cytometry analyses were performed on an LSR Fortessa X-20 flow cytometer (Becton Dickinson, New Jersey). The gating strategy was set up according to FMO control for all antibodies. Analysis and graphical output were performed using FlowJo™ software (Tree Star, Ashland, OR).

### Cell Culture and stimulation

Bone marrow-derived macrophages (BMDMs) were generated by differentiating mouse bone marrow cells for 10 days in DMEM medium (Life Technologies) supplemented with 10% heat-inactivated fetal calf serum (FCS, Life Technologies, Massachusetts), 100 U/mL penicillin, 100 µg/mL streptomycin (Gibco, Australia), 2 mM glutamine (Sigma-Aldrich, Missouri), 20% horse serum, and 30% (v/v) L929 conditioned medium as a source of M-CSF. On day 10, BMDMs were seeded in 24 wells plate at 10^6^ cells/well and stimulated with cGAMP (14 µM) or diABZI (0.3 and 1 µM) in DMEM containing 0.2% heat-inactivated FCS.

Human airway epithelial cells (hAEC, Epithelix, Switzerland), passage 1, isolated from bronchial biopsies were maintained in 75 cm^2^ flasks in the hAEC growth medium (Epithelix) and incubated at 37 °C and 5% CO2. hAEC (passage 8) were seeded in 24 wells plate at 5 × 10^5^ cells/well and stimulated after 4 h when they reached 80% confluence. Cells were stimulated with diABZI (1; 3 and 10 µM) and 2′,3′-cGAMP (14 µM) diluted in DMEM (Life Technologies, Massachusetts) supplemented with 2 mM l-glutamine, 2 mM of sodium pyruvate, 100 U/mL penicillin, 100 μg/mL streptomycin (Life Technologies), and 0.2% heat-inactivated FCS for 24 h at 37 °C and 5% CO2.

### Double-stranded DNA measurement in BALF

dsDNA was measured in the acellular fraction of the BALF using Quant-iT PicoGreen (Invitrogen, Massachusetts) according to the manufacturer’s protocol.

### Quantification of mitochondrial versus nuclear DNA

Mitochondrial and nuclear DNA were quantified as described previously [10]. Briefly, total DNA released in the BALF was purified using NucleoSpin Tissue Genomic DNA from tissue (Macherey-Nagel) and quantified by real-time PCR (10 ng/well). Primers for mouse mtDNA (mMitoF1 and mMitoR1) and mouse B2M (mB2MF1 and mB2MR1) that do not co-amplify nuclear mitochondrial insertion sequences (NumtS), mouse fragments of the mitochondrial genome present in the nuclear genome in the form of pseudogenes, were used [29]. The quantitative real-time PCR were performed in AriaMx Real-Time PCR System.

### Lactate dehydrogenase (LDH) measurement in BALF

Cytotoxicity was determined by quantifying lactate dehydrogenase (LDH) released in the acellular fraction of the BALF using LDH-Glo^TM^- Cytotoxicity assay kit (Promega) according to the manufacturer’s instructions.

### Quantification of mRNA expression by RT-qPCR analysis

Total mRNA was extracted using TRIzol (TRI-Reagent, Sigma-Aldrich, Germany) and reverse transcribed in cDNA with GoScript™ Reverse Transcription kit (Promega, Wisconsin). Genes mRNA expression were analyzed using GoTaq^®^qPCR Master Mix (Promega). All primer sequences used were from Qiagen: *Tmem173* (#QT00261590), *Mb21d1* (#QT00131929), *Nlrp3* (#QT00122458), *Ifi204* (#QT01753535), *Ddx41* (#QT00137130), *Aim2* (#QT00266819), *Cxcl10* (#QT00093436), *Ifna2* (*#QT00253092*)*, Ifna4* (*#QT01774353*), and *Ifnβ1* (#QT00249662). RNA expression was normalized to *Rn18s* expression (Qiagen, Maryland). Data were analyzed using the comparative analysis of relative expression by ^ΔΔ^Ct methods.

### Histology

Lung left lobe was removed and fixed in 4% formalin, embedded in paraffin, sectioned at 3 µm, stained with periodic acid-Schiff (PAS), and blindly scored by an anatomo-pathologist. Semi-quantitative scoring (0–5) of airway inflammation and damage including epithelial injury, peribronchial infiltrates, alveolitis, and emphysema severity was performed.

### Measurement of cytokine levels

MPO, IL-6, TNFα, CXCL1, IL-10, and CXCL10 concentrations in BALF or cell culture supernatant were measured by ELISA (R&D System, Minneapolis). IFNα and IFNβ levels were measured in BALF or cell culture supernatant by multiplex immunoassay according to manufacturers’ instructions (ProcartaPlex, Life Technologies, Massachusetts). Data were acquired on Luminex equipment (MagPix, Bio-Rad, California) and analyzed using Bioplex Manager software (Bio-Rad).

### Statistical analysis

Statistic analysis was performed with GraphPad Prism 8.0 software (San Diego). Statistical significance was determined by one-way ANOVA followed by Kruskal–Wallis multiple-comparisons tests or two-way ANOVA followed by Dunn post test as indicated in figure legends. *P* value < 0.05 was considered significant. *p < 0.05, **p < 0.01, ***p < 0.001 and ****p < 0.0001. All data were shown as mean ± SEM.

## Supplementary information

Supplemental Material and Figures

Article checklist

## Data Availability

All datasets generated and analysed during this study are included in this published article and its Supplementary Information files. Additional data are available from the corresponding author on reasonable request.
