# Supplementary material for: STING agonist diABZI induces PANoptosis and DNA mediated acute respiratory distress syndrome (ARDS)
Source: Cell Death Dis. 2022 Mar 25;13(3):269. doi: 10.1038/s41419-022-04664-5 (PMC8953969; doi:10.1038/s41419-022-04664-5)
Supplement: Supplementary file 1 — Supplemental Material and Figures [file 41419_2022_4664_MOESM1_ESM.pdf]

Supplemental Figure S1 *related to Figure 1*

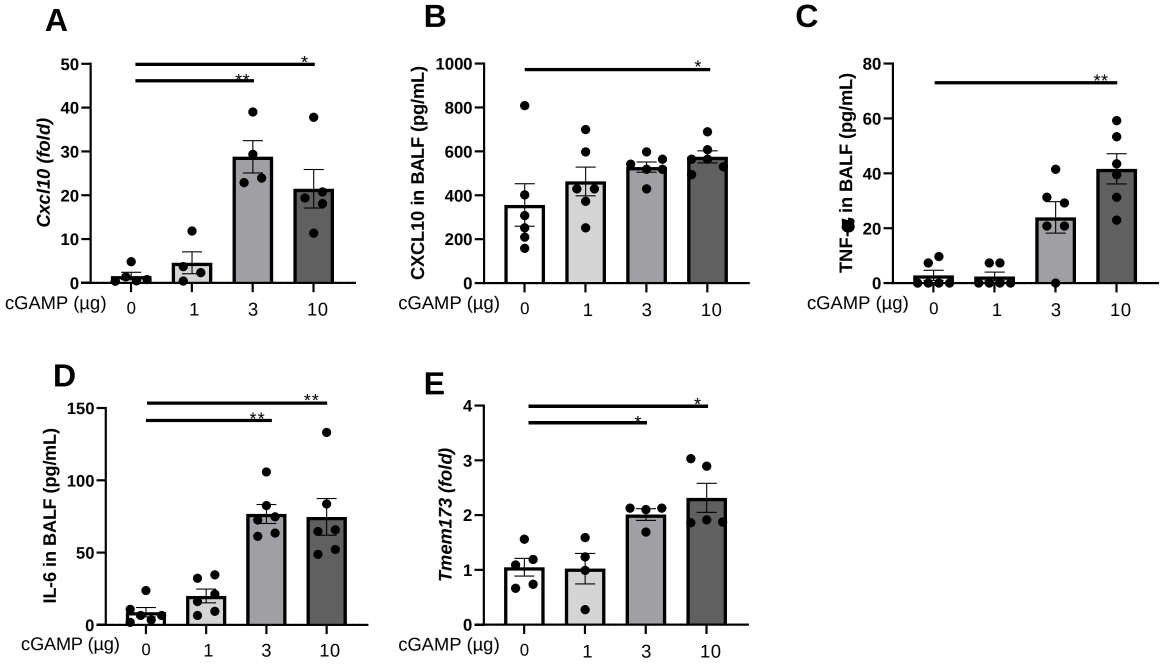

Supplemental Figure S1. cGAMP induced airway inflammation.

cGAMP (1, 3 or 10 µg, i.t.) or saline were administered daily in WT mice for 3 consecutive days and parameters analyzed on day 4 as desbribed in Figure 1. **A**-**B**. *Cxc10* transcripts (**A**), measured by real-time PCR and concentration of CXCL10 in BALF determined by ELISA (**B**), concentration of TNFα (**C**) and IL-6 (**D**) in BALF determined by ELISA. **D**. *Tmem173* transcripts measured by real-time PCR. Data are presented as mean ± SEM with n= 5-6 mice per group. Each point represents an individual mouse. *p < 0.05, **p < 0.01, ***p < 0.001, ****p < 0.0001 (Non-parametric Kruskal–Wallis test followed by Dunn post-test).

Supplemental Figure S2 *related to Figure 3*

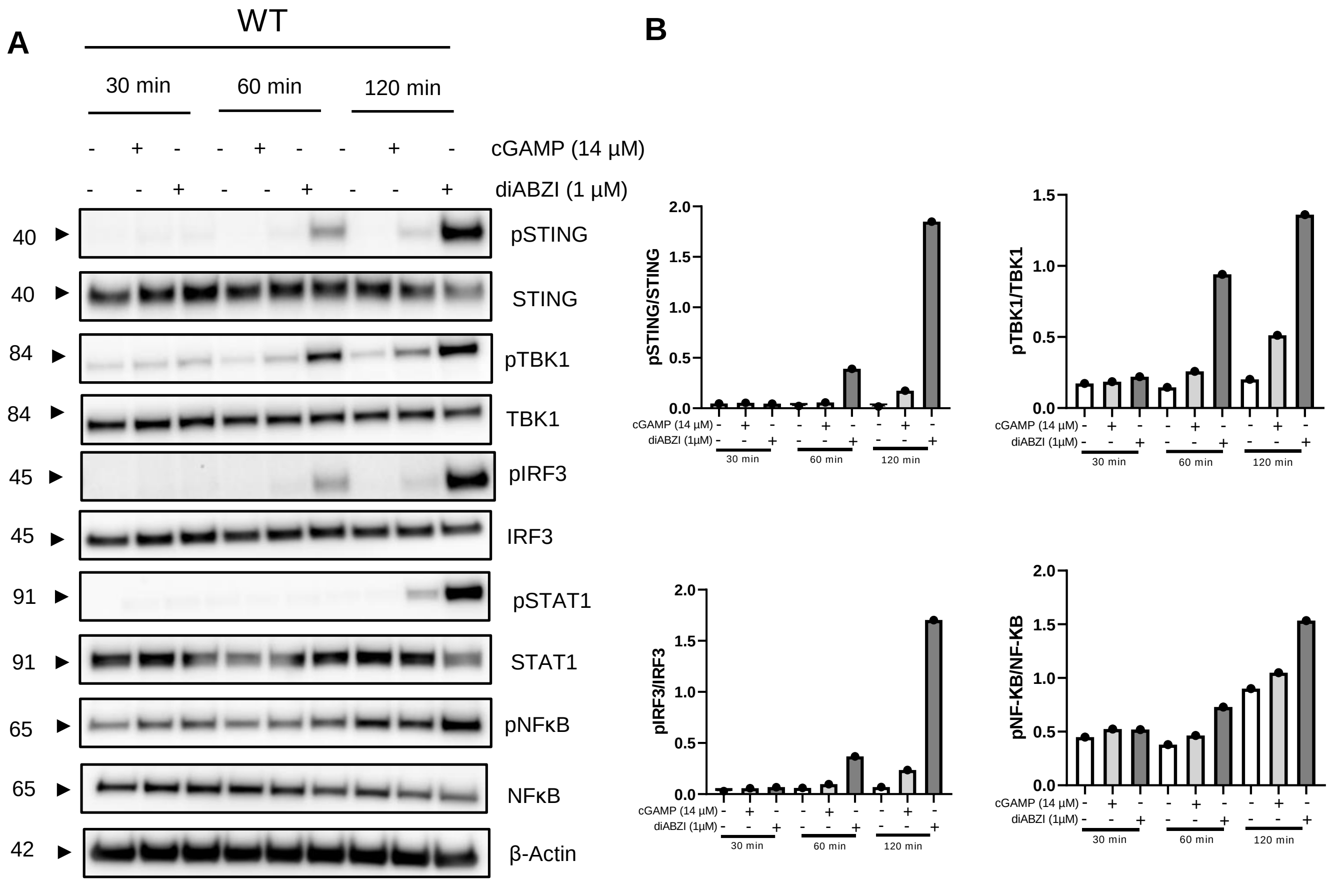

Supplemental Figure S2. diABZI induced STING axis activation at an early time-point.
A. Kinetic analysis of STING axis and NFkB axis by immunoblot at 30 min, 60 min and 120 min post-stimulation with cGAMP and diABZI, including phospho-STING, STING, phospho-TBK1, TBK1, phospho-IRF3, IRF3, phospho-STAT1, STAT1, phospho-NFkB, NFkB with β-actin obtained from BMDM lysate. **B.** Immunoblot quantification of pSTING, pIRF3, pTBK1 and pNFKB normalized to their total form.

Supplemental Figure S3 *related to Figure 6*

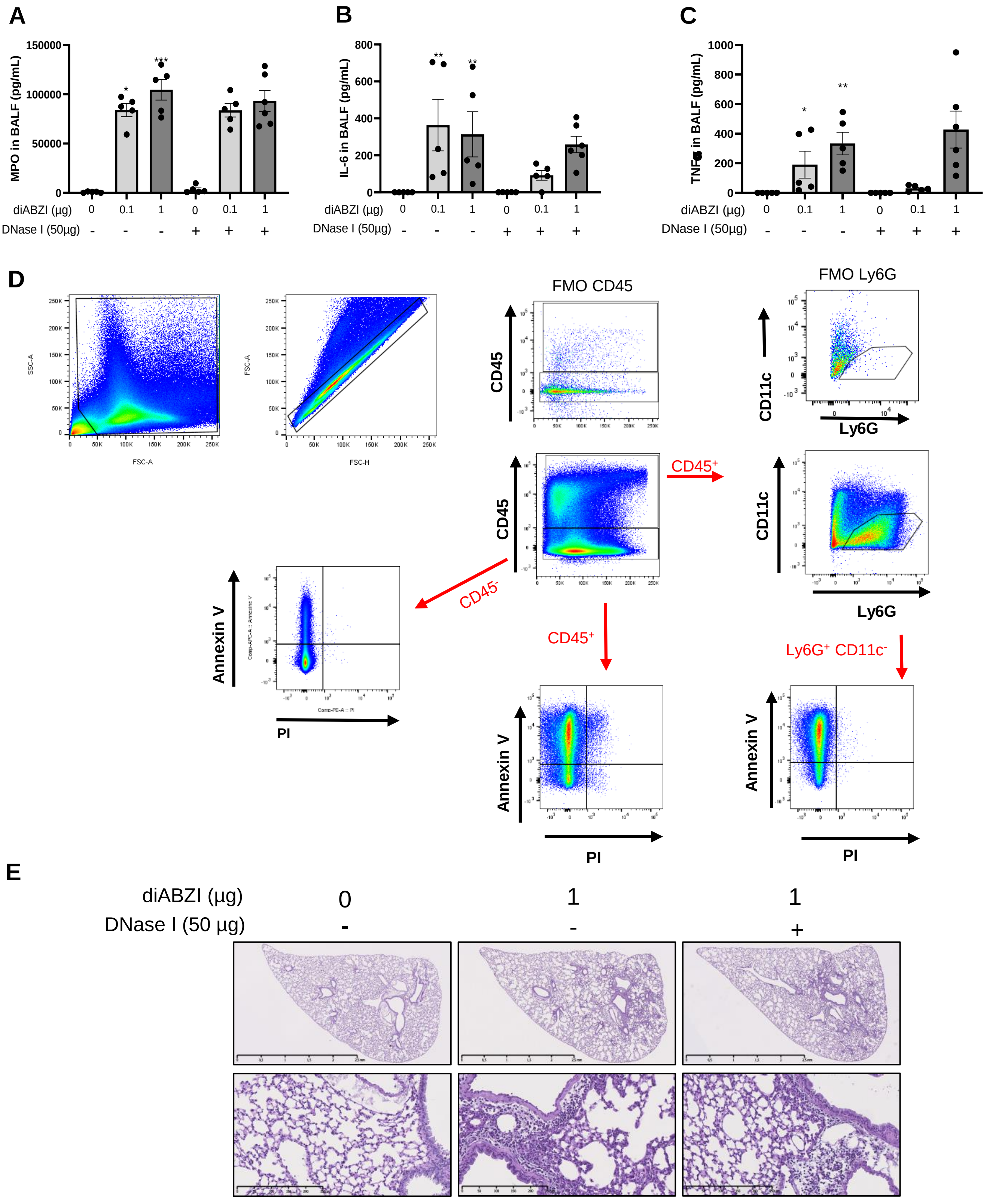

Supplemental Figure S3. DNase I treatment reduced diABZI induced airway inflammation.

diABZI (0.1 or 1 µg, i.t.) were administered with DNase I (50µg/mouse, i.t.) daily in WT mice for 3 consecutive days and parameters analyzed on day 4. **A-C**, Concentration of MPO (**A**), IL-6 (**B**) and TNFα (**C**) in BALF, determined by ELISA. **D**, gating strategy of Annexin V/PI staining of pre-gated singlets (SSC-A/SSC-H), $CD45^{+}$ (leukocytes) and $CD45^{+}Ly6G^{+}CD11c^{-}$ cells (neutrophils). **E**, Lung tissue histology PAS staining. Bars, upper panels: 2.5 mm, lower panels: 250 µm. Data are presented as mean ± SEM with n= 5-6 mice per group. Each point represents an individual mouse. *p < 0.05, **p < 0.01, ***p < 0.001, ****p < 0.0001 (Non-parametric Kruskal–Wallis test followed by Dunn post-test).

Supplemental. Figure S4 related to Figure 7

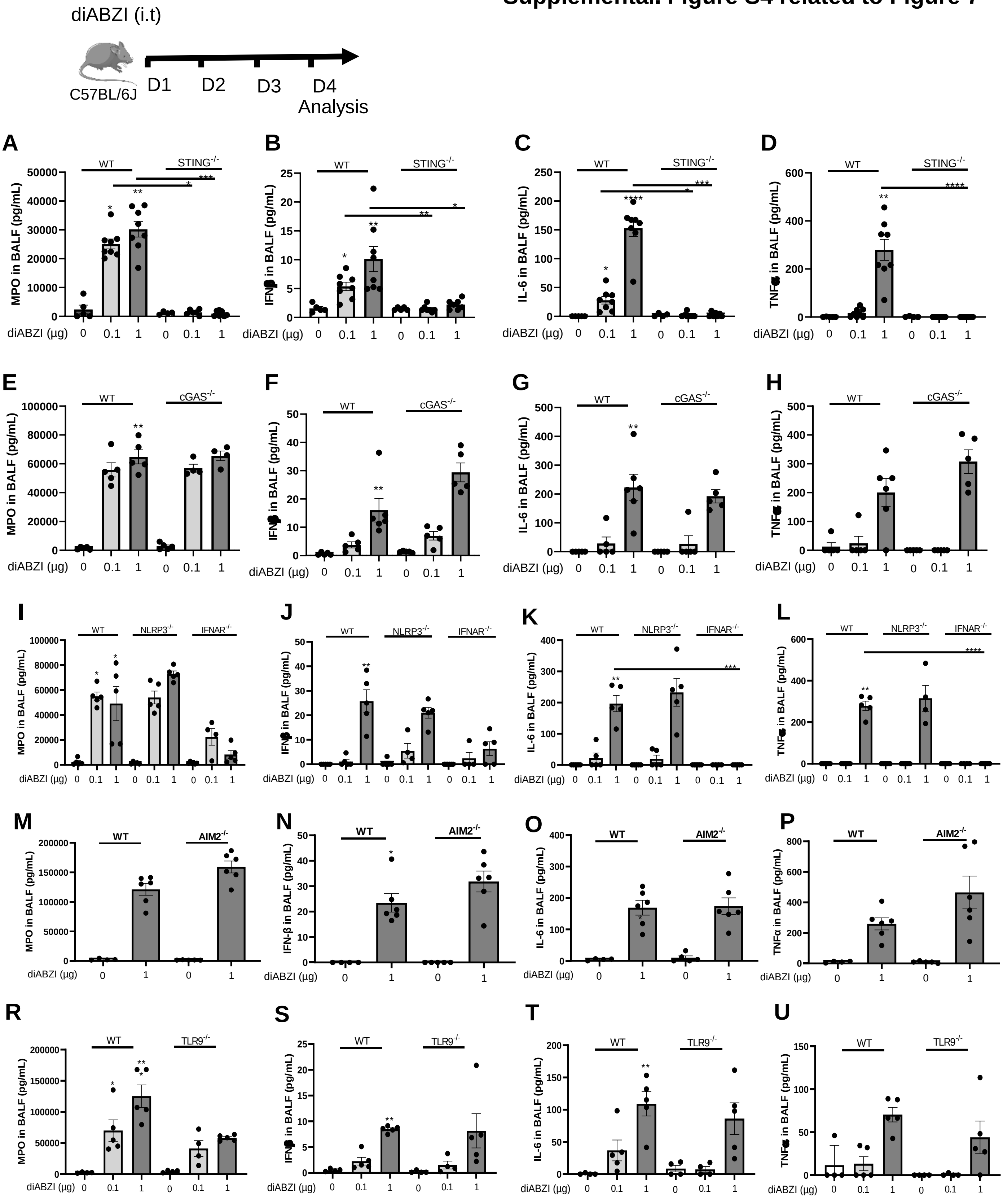

Supplemental Figure S4. STING is a major sensor of diABZI induced lung inflammation

DiABZI (0.1 or 1 µg, i.t.) or saline were administered daily in STING$^{-/-}$, cGAS$^{-/-}$, NLRP3$^{-/-}$, IFNAR$^{-/-}$, TLR9$^{-/-}$, AIM2$^{-/-}$ and WT mice for 3 consecutive days and parameters analyzed on day 4. **A-D**. Concentrations of MPO (**A**), IFNβ (**B**), IL-6 (**C**) and TNFα (**D**) in BALF in WT and STING$^{-/-}$ mice. **E-H**. Concentrations of MPO (**E**), IFNβ (**F**), IL-6 (**G**) and TNFα (**H**) in BALF in WT and cGAS$^{-/-}$ mice. **I-L**. Concentrations of MPO (**I**), IFNβ (**J**), IL-6 (**K**) and TNFα (**L**) in BALF in WT and NLRP3$^{-/-}$ and IFNAR$^{-/-}$ mice. **M-P**. Concentrations of MPO (**M**), IFNβ (**N**), IL-6 (**O**) and TNFα (**P**) concentration in BALF in WT and AIM2$^{-/-}$ mice. **R-U**. Concentrations of MPO (**R**), IFNβ (**S**), IL-6 (**T**) and TNFα (**U**) in BALF in WT and TLR9$^{-/-}$ mice. Graph data are presented as mean ± SEM with n=5-6 mice/group. Each point represents an individual mouse. *$p < 0.05$, **$p < 0.01$, ***$p < 0.001$. (Non-parametric Kruskal–Wallis test followed by Dunn post-test).

Supplemental Figure 5: full Western blots

Supplemental Figure S5A *related to Figure 1O*

cGAMP *in vivo*

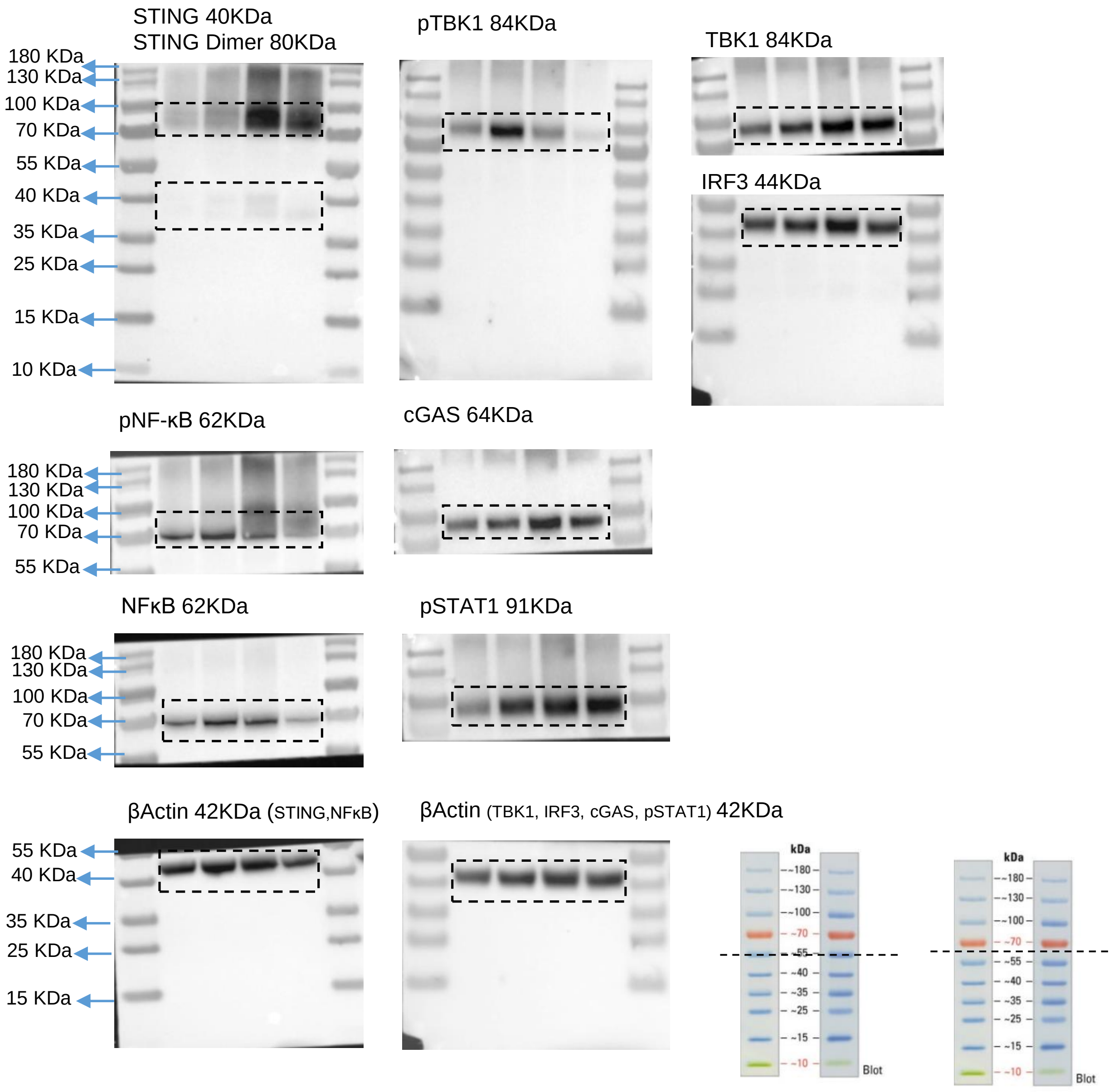

Supplemental Figure S5. Uncropped immunoblots of the different figures

A. Immunoblots related to Figure 1O, immunoblots of STING Dimers, STING, phospho-TBK1, TBK-1, IRF3, cGAS, pNF-κB, NF-κB, pSTAT1 with β-actin obtained from lungs tissu lysate.

Supplemental Figure 5: full Western blots

Supplemental Figure S5B *related to Figure 3H-I*

Macrophages (BMDM)

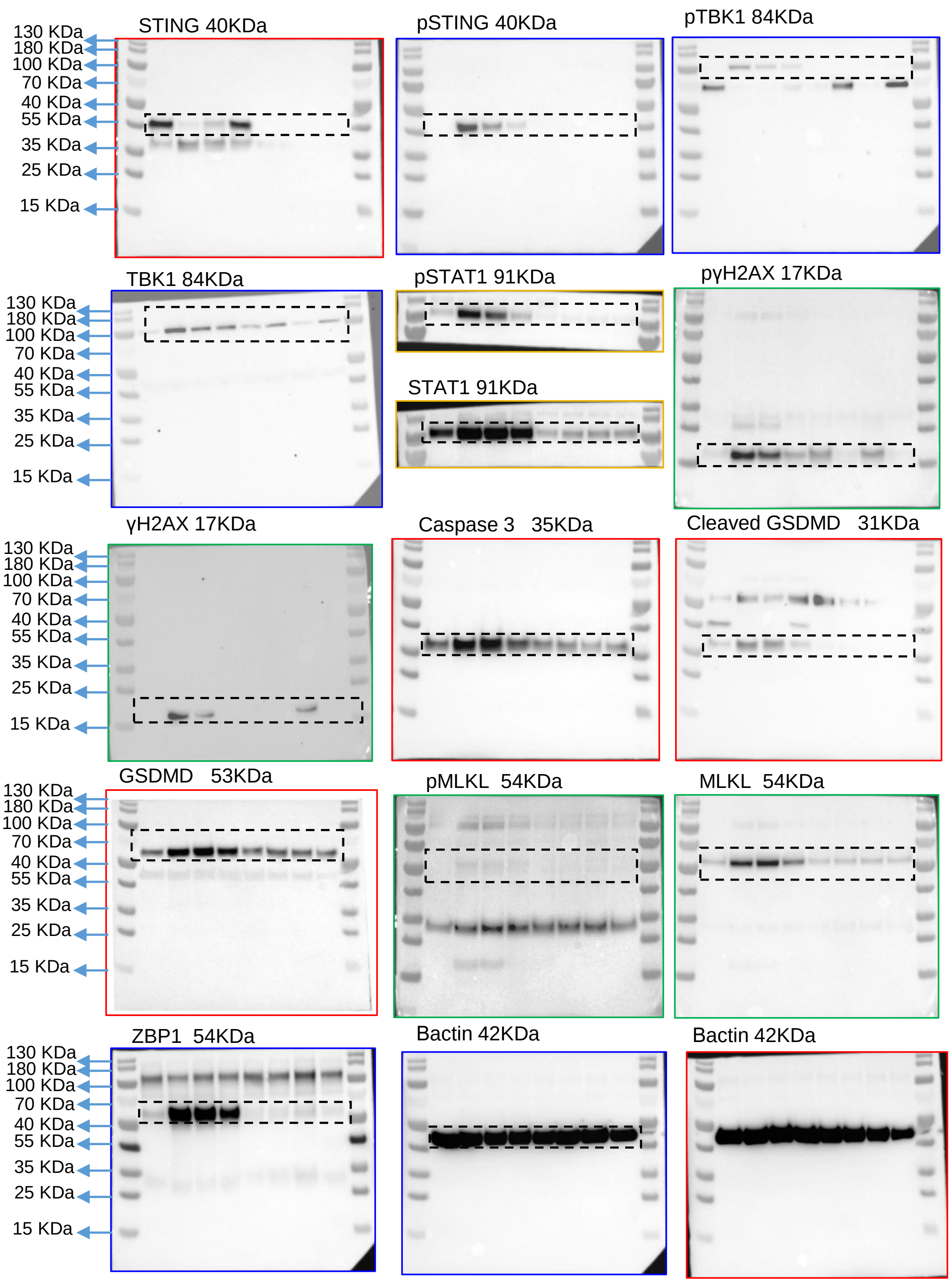

Supplemental Figure S5B *related to Figure 3H-I*

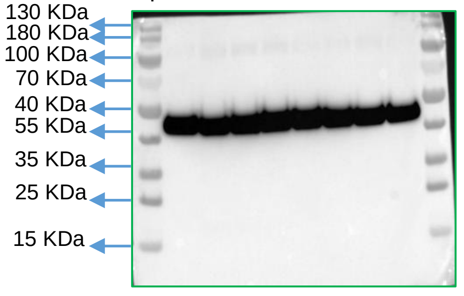

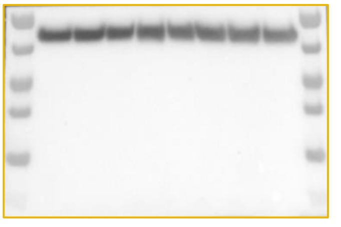

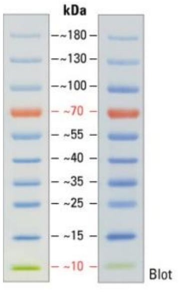

Supplemental Figure S5. Uncropped immunoblots of the different figures

B. Immunoblots related to **Figure 3H,** immunoblots of STING, phospho-STING, phospho-TBK1, TBK1, phospho-STAT1, STAT1, phospho-γH2AX, γH2AX**. Figure 3I,** immunoblots of Caspase-3, Cleaved GSDMD, GSDMD, phospho-MLKL, MLKL, ZBP1 with β-actin obtained from BMDM lysate**.**

Supplemental Figure S5C *related to Figure 3M*

Human airway epithelial cells (hAEC)

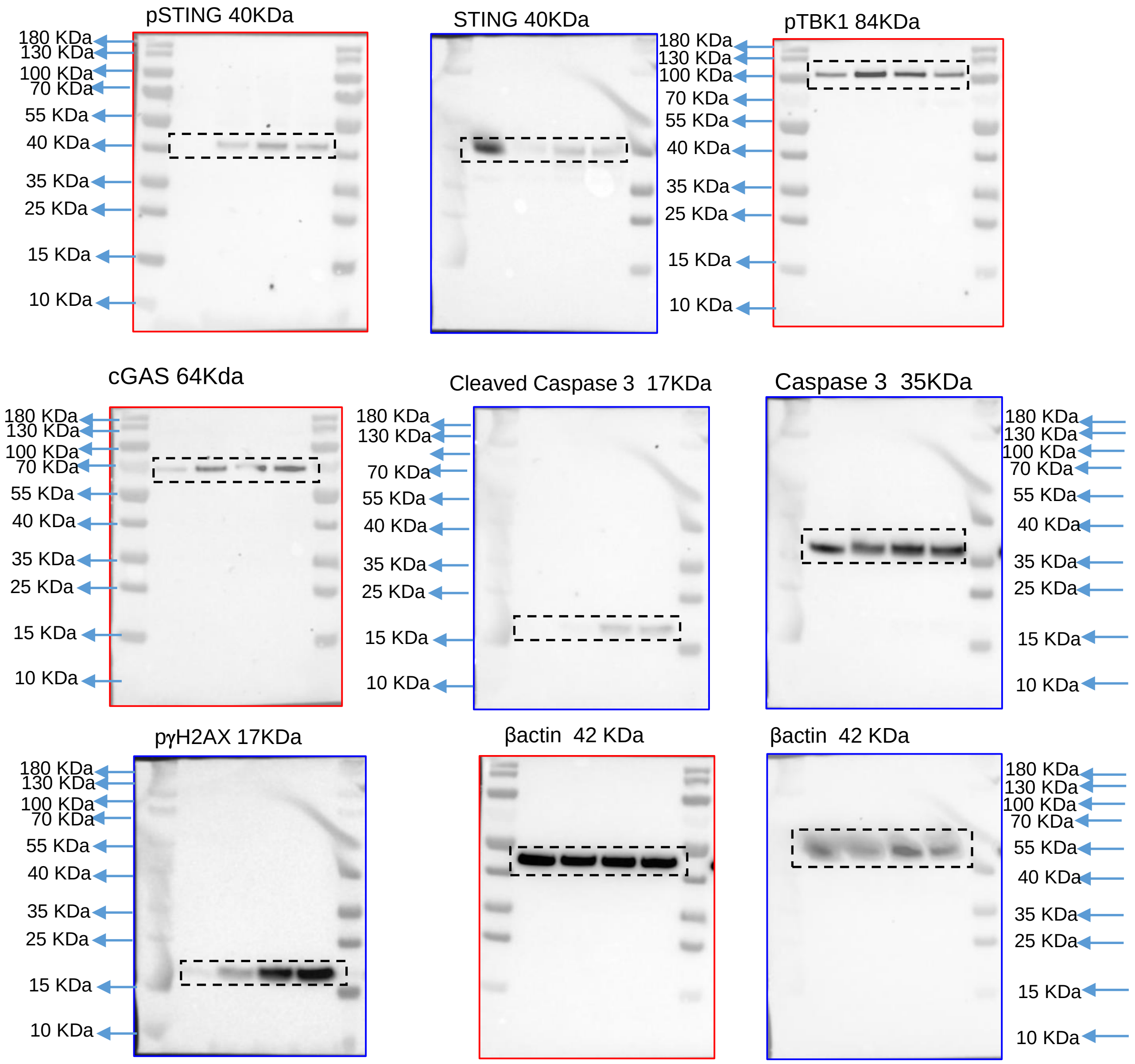

Supplemental Figure S5C Uncropped immunoblots of the different figures

C. Immunoblots related to Figure 3M, immunoblots of phospho-STING, STING, phospho-TBK1, cGAS, Cleaved caspase-3, Caspase-3, phospho-γH2AX, with β-actin obtained from human airway epithelial cells lysate.

Supplemental Figure S5D *related to Figure 5A*

In vivo

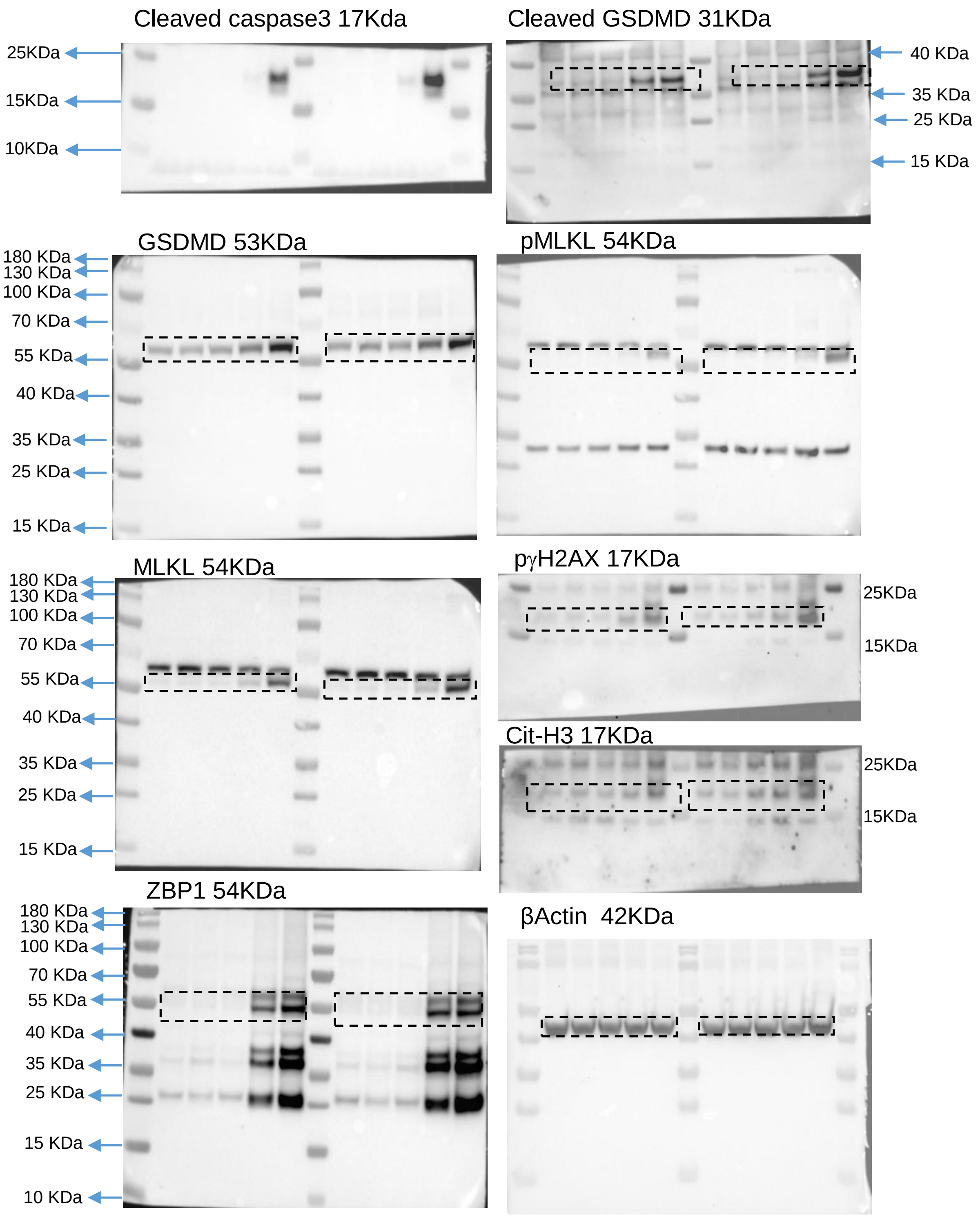

Supplemental Figure S5D Uncropped immunoblots of the different figures

D. Immunoblots related to Figure 5A, immunoblots of cleaved caspase-3, cleaved GSDMD, GSDMD, phospho-MLKL, MLKL, phospho-γH2AX, Cit-H3, ZBP1 with β-actin obtained from lungs tissue lysate.

Supplemental Figure S5E *related to Figure 5D*

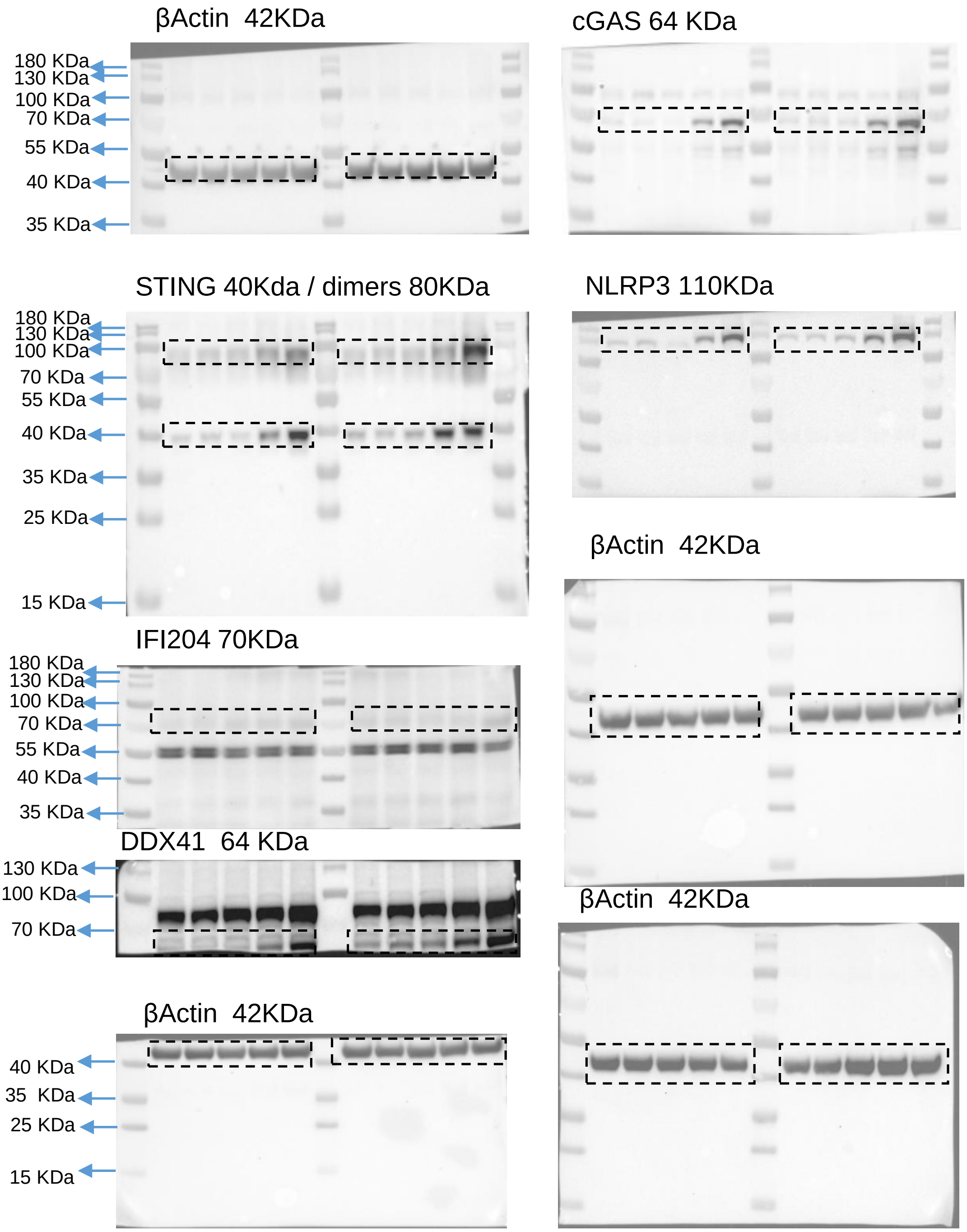

Supplemental Figure S5E Uncropped immunoblots of the different figures
E. Immunoblots related to Figure 5D, immunoblots of cGAS, STING, NLRP3, IFI204, DDX41 with β-actin obtained from lungs tissue lysate.

Supplemental Figure S5F *related to Figure 6N*

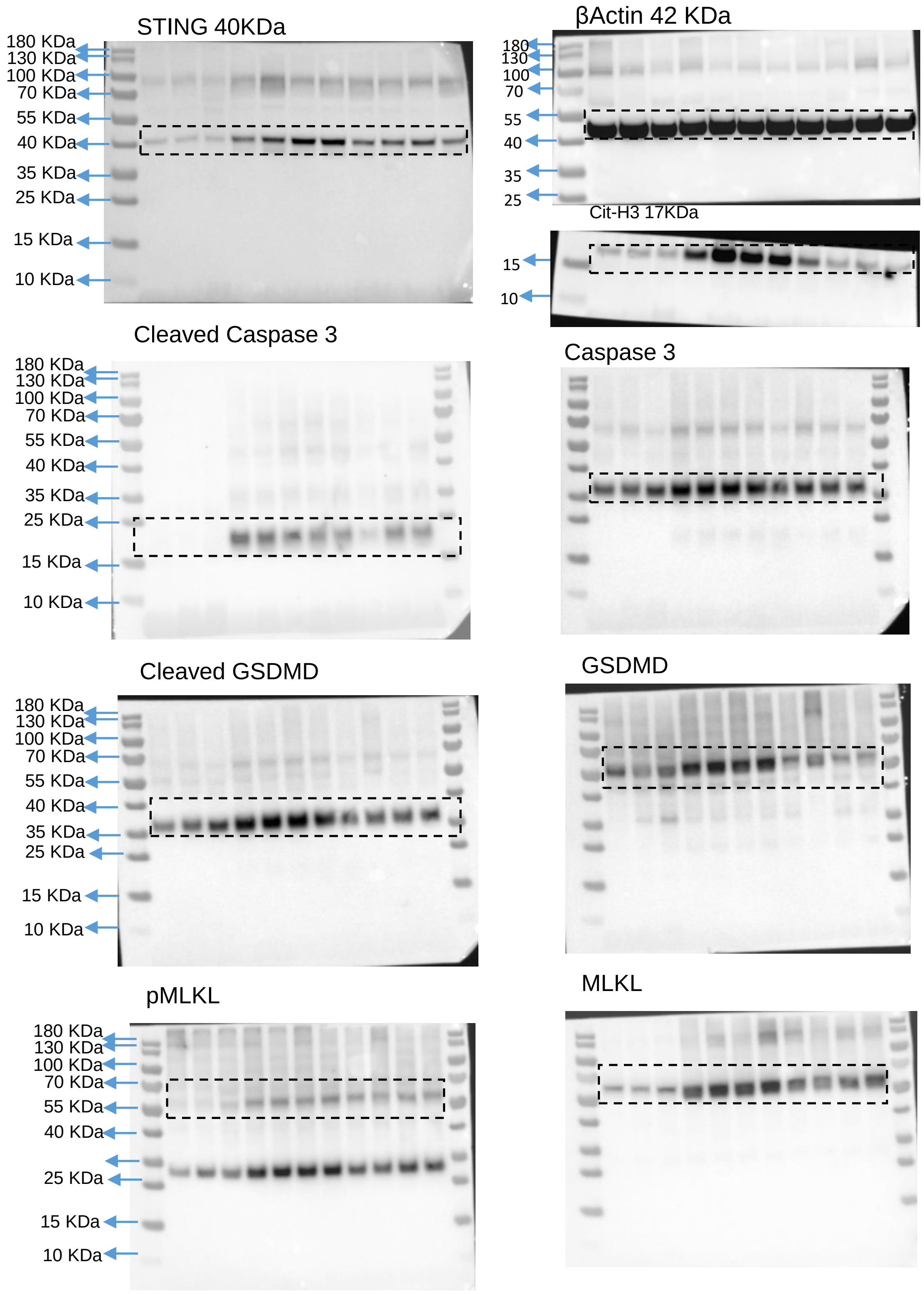

Supplemental Figure S5F *related to Figure 6N*

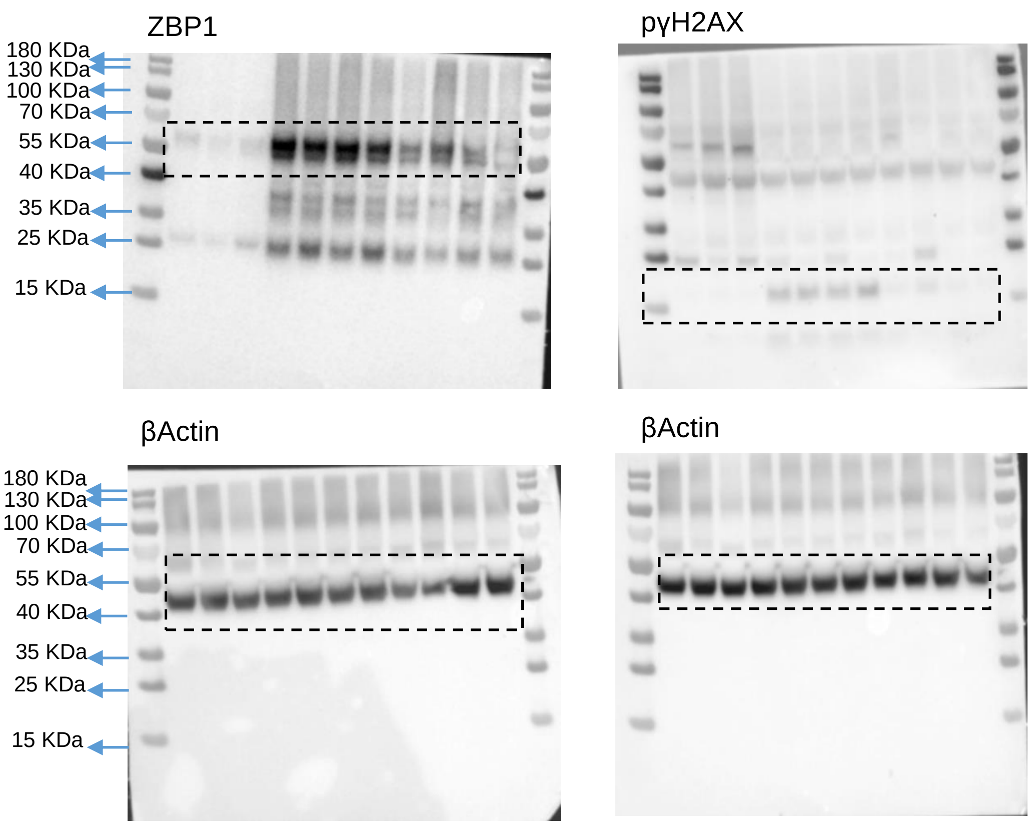

Supplemental Figure S5F Uncropped immunoblots of the different figures

F. Immunoblots related to Figure 6N, immunoblots of Cit-H3, STING, Cleaved caspase-3, Caspase-3, Cleaved-GSDMD, GSDMD, phospho-MLKL, MLKL, phospho-γH2AX, ZBP1 with b-actin obtained from lungs tissue lysate.

Supplemental Figure S5G *related to Figure S2*

BMDM

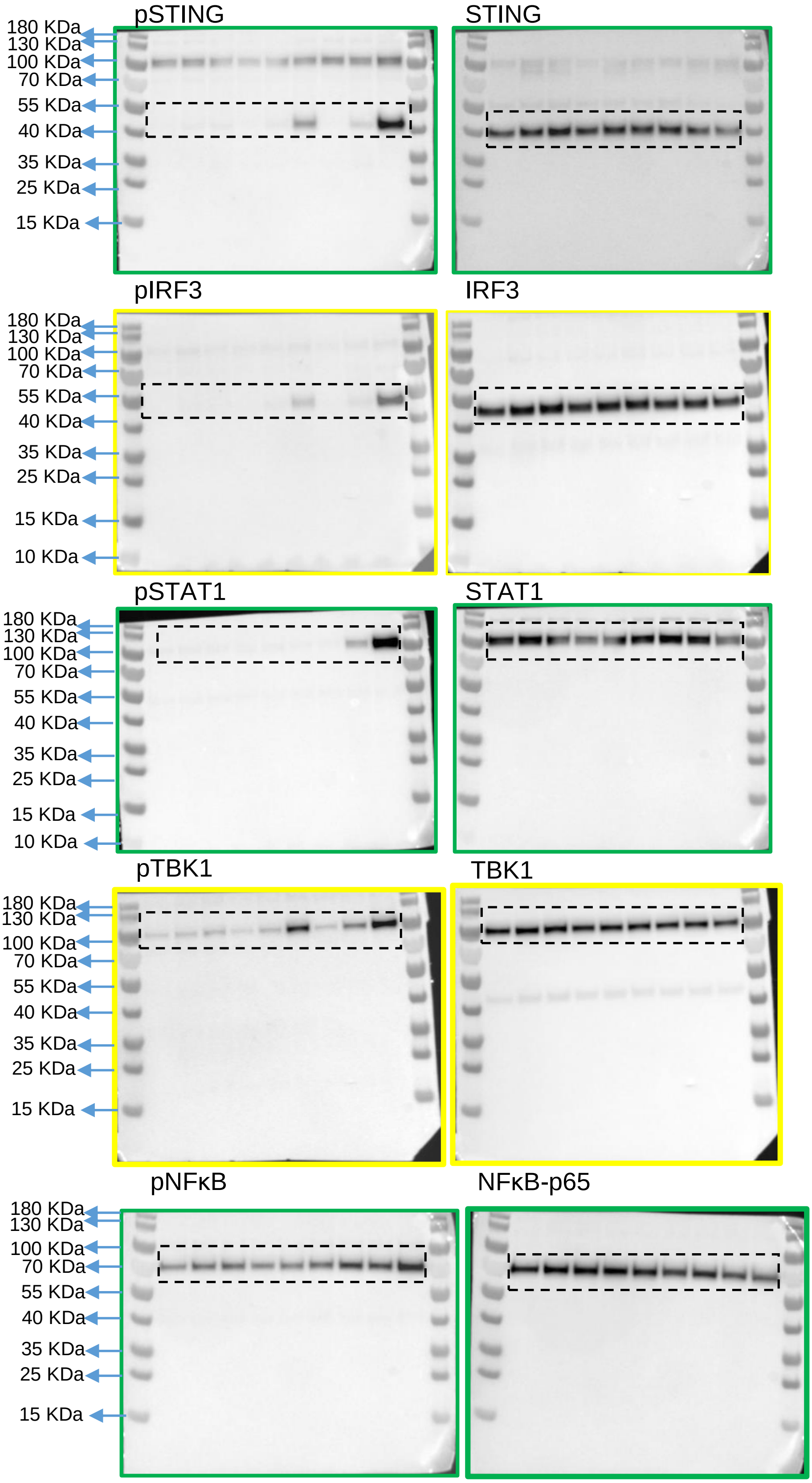

Supplemental Figure S5G *related to Figure S2*

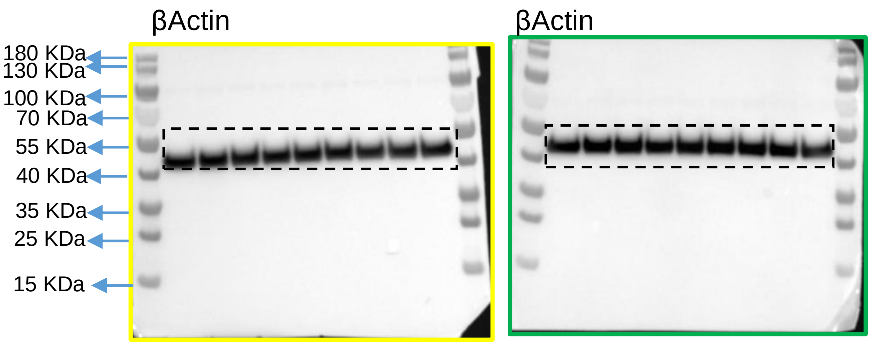

Supplemental Figure S5G Uncropped immunoblots of the different figures
G. Immunoblots related to Figure S2, immunoblots of phospho-STING, STING, phospho-IRF3, IRF3, phospho-STAT1, STAT1, phospho-TBK1, TBK1, phospho-NFkB, NFkB with β-actin obtained from BMDM lysate.
